# Differences in functional traits and drought tolerance between heteromorphic leaves of *Artemisia tridentata* seedlings, a keystone species from a semiarid shrubland

**DOI:** 10.1093/aobpla/plaf051

**Published:** 2025-09-14

**Authors:** Marcelo Serpe, Jacob Venable, Sven Buerki

**Affiliations:** Department of Biological Sciences, Boise State University, 1910 University Drive, Boise, ID 83725-1515, United States; Department of Biological Sciences, Boise State University, 1910 University Drive, Boise, ID 83725-1515, United States; Department of Biological Sciences, Boise State University, 1910 University Drive, Boise, ID 83725-1515, United States

**Keywords:** adaptive plasticity, drought responses, native plants, seedling establishment

## Abstract

Leaf traits are crucial to seedling growth and survival, and their plasticity can influence seedling fitness in changing environments. Seedlings of *Artemisia tridentata*, a keystone shrub of the western North American sagebrush steppe, show heteromorphic leaf development. Early leaves are larger and less pubescent than those produced later, suggesting a shift from characteristics favouring rapid growth to those increasing drought tolerance. To investigate this hypothesis, we determined the specific leaf area (SLA) and the osmotic potential at full turgor (π_0_) of early and late leaves, and measured their stomatal conductance and photosynthetic rates as leaf water potential (Ψ_l_) declined under imposed drought. We also examined whether water stress could trigger late leaf development. At high Ψ_l_ and per area, early and late leaves had similar photosynthetic rates. However, the SLA of early leaves was three times higher than that of late leaves, yielding higher photosynthetic rates per unit mass in the former. Late leaves had lower π_0_ and were less sensitive to drought, exhibiting a lower Ψ_l_ at 50% of maximum photosynthesis than early leaves. Drought triggered the shedding of early leaves and the initiation of late-like leaves. Formation of these leaves continued upon return to well-watered conditions, possibly indicating stress memory. The overall results suggest that early leaves enhance growth during wet springs following germination, while late leaves prolong photosynthesis as water potentials decline during summer drought. The adaptive value of early leaves may be diminishing due to changing environmental conditions that are accelerating the onset of drought.

## Teaser

Seedlings of the drought-tolerant shrub big sagebrush (Artemisia tridentata) show leaves with different morphologies. Early leaves are larger and less pubescent than those produced later, suggesting a shift from characteristics favoring rapid growth to those increasing drought tolerance. We investigated this hypothesis by measuring photosynthesis under imposed drought and analyzing various leaf traits. Early leaves of well-watered seedlings had higher photosynthetic rates per unit mass than late leaves. However, as drought developed, the decline in photosynthesis was more gradual in late leaves. In young seedlings, drought triggered shedding of early leaves and initiation of late-like leaves. Formation of these leaves continued upon rewatering, possibly indicating stress memory. Our results suggest that early leaves enhance growth during wet springs following germination, while late leaves prolong photosynthesis during summer drought. The adaptive value of early leaves may be diminishing.

## Introduction

Various plant species show marked differences in leaf morphology within an individual plant (Winn 1999, Zotz et al. 2011, Nakayama et al. 2017). Such differences can result from ontogenic transitions in the shoot meristems, known as heteroblasty, or from plant exposure to different environmental conditions, referred to as heterophylly (Zotz et al. 2011, Chitwood and Sinha 2016, Nakayama et al. 2017). Heteroblasty and heterophylly occur in distantly related taxa, suggesting that these traits have evolved independently multiple times (Chen et al. 2011, Leigh et al. 2011, Nakayama et al. 2017, Li et al. 2019). From a functional perspective, heteroblasty and heterophylly are thought to enhance plant fitness in coping with environmental heterogeneity (Winn 1999, Wells and Pigliucci 2000, Nakayama et al. 2017).

Heterophylly can be triggered by environmental differences surrounding an individual plant or by changes occurring at a larger spatial or temporal scale (De Kroon et al. 2005). The first situation is well-documented for heterophyllous aquatic and amphibious plants, where the morphology and anatomy of submerged leaves markedly differ from those of aerial ones (Wells and Pigliucci 2000, Nakayama et al. 2017). On a larger scale, such as affecting the entire shoot of many plants, heterophylly can be caused by seasonal climate changes (Mulkey et al. 1992). For instance, semideciduous shrubs and trees growing in savannas and Mediterranean biomes often show heteromorphic leaves (Palacio et al. 2006, Rossatto and Kolb 2009). Typically, larger and thinner leaves are present under mesic conditions, while smaller and thicker leaves become dominant during periods of water stress (Westman 1981, Aronne and De Micco 2001, Palacio et al. 2006).

A seasonal dimorphic pattern of leaf development also occurs in a keystone species of the sagebrush steppe of western North America, the shrub *Artemisia tridentata* Nutt (Asteraceae; big sagebrush). Miller and Shultz (1987) described the phenology of leaf development for one of the subspecies of big sagebrush, *A. tridentata* ssp. *wyomingensis*. In this subspecies, larger leaves emerge in late winter and early spring, while smaller ones develop throughout the rest of the spring and early summer. The larger leaves are called ephemerals because most fall during the summer with the onset of drought (Miller and Shultz 1987). Instead, the smaller leaves remain during the summer, fall, and winter. Due to this characteristic, they are known as persistent, although they begin to drop after 12–13 months (Miller and Shultz 1987). A recent study reported similar phenologies for ephemeral and persistent leaves of another *A. tridentata* subspecies, *A. tridentata* ssp. *tridentata* (Heil et al. 2025). Still, information about the longevity of ephemeral and persistent leaves is scarce. Given the high genetic diversity of *A. tridentata* and the vast geographical area where it grows, the longevity of ephemerals and persistent leaves may vary between genotypes and the specific environment in which the plants grow (Richardson et al. 2012, Doherty et al. 2024).

**Figure 1.**
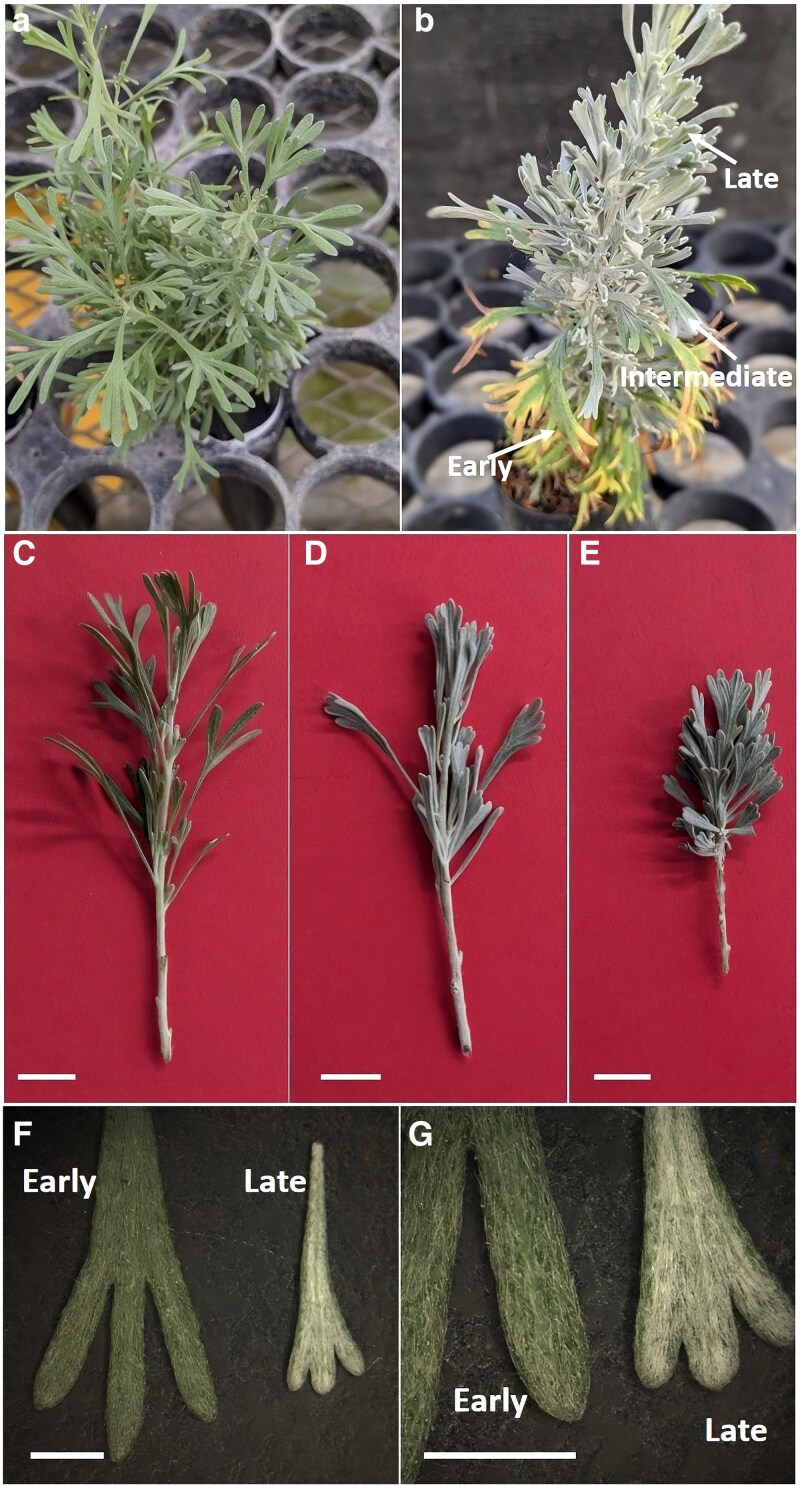
Differences in leaf characteristics in *Artemisia tridentata* seedlings. Examples of a 3-month-old seedling with early leaves (a) and a 9-month-old seedling (b) with early, intermediate, and late leaves. Terminal portions of shoots in 3- (c), 6- (d), and 12-month-old (e) seedlings showing differences in colour, size, and degree of dissection between early (c), intermediate (d), and late (e) leaves. Closer view of early and late leaves to illustrate their difference in trichome density (f and g). Bars = 1 cm (c, d, and e), 3 mm (f and g).

Although, to our knowledge, not documented, *A. tridentata* also shows heteromorphic leaves at the seedling stage. Our pictures illustrate that the leaves that form after the cotyledons have a relatively low trichome density and are markedly dissected (Fig. 1a, c, and f). Eventually, the seedlings develop leaves that are grayish due to a significantly higher trichome density, and these leaves are also shorter, less dissected, and more similar to those of adult plants (Fig. 1b, e and g). In addition, between these two stages, there are leaves with intermediate characteristics, more pubescent than early leaves and larger than late leaves (Fig. 1b and d). To distinguish the three general types of leaves present in the seedlings from each other and leaves in adult plants, we will refer to them as early, intermediate, and late leaves.

As shown for other species (Tripathi et al. 2020), in *A. tridentata,* changes in leaf characteristics at the seedling stage could play a significant role during plant establishment. The seeds of this plant germinate in late winter to early spring, when temperatures tend to be above 0°C and soil moisture is high due to snowmelt and precipitation (DiCristina and Germino 2006, Schlaepfer et al. 2014). During this period, a decrease in the number of days under these conditions can markedly decrease establishment (Brabec et al. 2017, O'Connor et al. 2020). As spring and summer progress, the seedlings become gradually more exposed to drought and heat, leading to decreases in water potential (DiCristina and Germino 2006, Schlaepfer et al. 2014, 2021). Under these contrasting environments, seedlings would benefit from high carbon assimilation, rapid growth, and the development of an adequate root system during periods with high moisture (Lazarus et al. 2019), while switching to a more water-conserving strategy during the summer. Leaf traits can influence growth rates, and there are trade-offs between structural and functional characteristics that favour growth and those that confer cold and drought tolerance (Falster et al. 2018, Lazarus et al. 2019, Guillemot et al. 2022, Kaproth et al. 2023). For *A. tridentata* seedlings, a relevant question addressed in the present study is the extent to which differences in leaf morphology are associated with a trade-off between maximizing carbon gain and remaining functional as drought develops.

Studies on seedlings of *A. tridentata* are of significant interest due to the ecological importance of this species and the threats it faces (Applestein et al. 2021, Schlaepfer et al. 2021). This dominant shrub contributes to the development of a heterogeneous landscape and provides habitat and forage for local animals, including obligate fauna (Charley and West 1977, Davies et al. 2007, Larrucea and Brussard 2008, Homer et al. 2015). Over the past century, sagebrush habitats have been lost to agriculture and urban development and have been disturbed by overgrazing and invasion by exotic annual grasses (Knick et al. 2003, Shi et al. 2018, Schlaepfer et al. 2021). This invasion has significantly impacted sagebrush communities by increasing wildfire frequency, which tends to eliminate *A. tridentata* and other native vegetation components (Dantonio and Vitousek 1992, Brooks et al. 2004). Efforts to restore *A. tridentata* have been extensive, and a recent analysis indicates that overall these efforts have improved reestablishment (Simler-Williamson and Germino 2022). Nevertheless, challenges exist in reestablishing *A. tridentata*, particularly in drier and warmer sites, as well as those that have experienced higher levels of disturbance (Schlaepfer et al. 2021, Simler-Williamson and Germino 2022). Knowledge of seedling adaptations and plastic responses can be valuable in improving reestablishment and predicting recruitment under current and anticipated climate change scenarios (O’Connor et al. 2020, Melton et al. 2023, Vasey et al. 2023).

To determine possible differences in growth potential and drought tolerance between early and late leaves, we examined the effect of decreases in leaf water potential (Ψ_l_) on transpiration and photosynthesis (Klein 2014, Herrera et al. 2022). In addition, we measured two functional traits in early, intermediate, and late leaves that provide complementary information: the specific leaf area (SLA) and the osmotic potential at full turgor (π_0_). SLA typically shows a positive relationship with growth, particularly in seedlings, while π_0_ is used to estimate the turgor loss point π_tlp_; lower (more negative) π_0_ and π_tlp_ values indicate a higher capacity to cope with drought (Bartlett et al. 2012b, Falster et al. 2018). We hypothesized that early leaves would have a higher capacity to support growth at high Ψ_l_, which would be indicated by higher photosynthetic rates and SLA than late leaves. In contrast, late leaves would be able to maintain photosynthesis to lower water potentials due to lower π_0_ and π_tlp_ than early leaves, signifying that late leaves are more drought-tolerant.

Another intriguing question regarding leaf changes with seedling development investigated in this study is what causes the switch from one form to another. Morphological differences between the leaves may be related to a transition from a juvenile to an adult phase, thus a heteroblastic change (Zotz et al. 2011, Manuela and Xu 2020). However, given that *A. tridentata* shows seasonally heteromorphic leaves in adult plants, it is also possible that environmental factors cause changes in morphology at the seedling stage, either directly or by affecting the timing of the juvenile-to-adult transition (Miller and Shultz 1987, Manuela and Xu 2020, Poethig and Fouracre 2024). In natural habitats, the developmental changes individuals experience co-occur with environmental changes (Miller and Shultz 1987, Heil et al. 2025). Consequently, it is difficult to distinguish the contribution of intrinsic ontogenic transitions from that of environmental factors to alterations in leaf development. To minimize the effect of environmental factors on leaf morphology and other traits, we grew seedlings under a consistent daily cycle of ambient conditions and a watering regimen that maintained the seedlings at high water potentials. A marked shift in leaf characteristics with seedling age under these rather uniform conditions would support the notion that endogenous developmental factors contribute to heteromorphic leaf development (Poethig and Fouracre 2024). Because the seedlings are naturally exposed to drought, we hypothesized that water stress would induce the transition from early to late-like leaves. To test this hypothesis, we exposed 2- and 4-month-old seedlings with mainly early leaves to water stress. One complicating factor in this analysis was that leaf expansion is one of the most, if not the most, sensitive processes to water deficits (Tardieu et al. 2011). Thus, smaller leaves under water stress could result from biophysical constraints caused by drought, such as a reduction in cellular turgor, rather than a genetically programmed change in leaf characteristics. To distinguish between these possibilities, we analysed leaf size, SLA, and π_0_ on leaves of water-stressed seedlings after they were returned to well-watered conditions from 1 to 3 months. We hypothesize that these leaves would have late-leaf characteristics. Such a result would suggest that water stress triggered transcriptomic changes that fixed leaf development to the drought state despite returning to moist conditions (Sadhukhan et al. 2022, Melton et al. 2023, Rahman et al. 2025).

## Materials and methods

### Plant material and growing conditions

In this study, we used *Artemisia tridentata* ssp. *wyomingensis* (Wyoming big sagebrush, hereafter referred to as *A. tridentata*) seeds collected from several mother plants at Kuna Butte, Idaho, USA (43°26.161′ N, 116°25.848′ W, 908 m a.s.l.). The seeds were planted in 150 ml cone-tainers (SC10R-Ray Leach, Stuewe & Sons, Inc., Tangent, OR, USA) filled with a 1:1 perlite-to-vermiculite mix. After germination, seedlings were thinned to one seedling per cone-tainer and grown in a greenhouse under a 14-hour photoperiod with day/night conditions of 23/18 ± 3°C. The greenhouse light intensity varied depending on the amount of sunlight, but generally ranged from 150 to 400 µmol m^−2^ s^−1^ of photosynthetically active radiation (PAR) during the light period. We watered the seedlings with a nutrient solution (Flora Series, Genhydro, Sebastopol, CA, USA) using an ebb-and-flow hydroponics system programmed to submerge the lower 10 cm of the cone-tainers for 30 minutes daily. In this sub-irrigation system, the nutrient solution was used at the recommended concentration for cutting and seedlings, and the watering schedule kept the mix close to the pot's capacity. We maintained the pH of the nutrient solution between 5.7 and 6.2 and replaced it monthly. Seedlings were grown under these conditions for various times depending on the sampling time and treatment (see below). The above experimental conditions were selected to minimize environmental variability, thereby facilitating the distinction between changes caused by intrinsic ontogenic processes from those triggered by environmental change. Lower temperatures at night and higher light intensities would have been preferable to mimic the natural environment better (Brabec et al. 2017, Geisler et al. 2022). However, maintaining lower temperatures was not possible with the available controls, and the supplemental lights provided only between 150 and 200 µmol m^−2^ s^−1^ of PAR and were mainly used to extend the photoperiod when necessary. The nutrient concentrations used are low for hydroponics but likely higher than those found in sagebrush steppe soils (Bates and Davies 2017, Velazquez-Gonzalez et al. 2022). We could have grown the seedlings under lower nutrient levels; however, this would have increased the risk of causing deficiencies, complicating the interpretation of the results.

### Leaf area, SLA, and osmotic potential at full turgor (π_o_) of early-, intermediate-, and late-leaves

Due to variations in shape, size, and pubescence, the classification of leaves as early, intermediate, and late is not very precise, particularly for intermediate leaves. To link more directly changes in leaf characteristics to seedling development, we sampled seedlings at four stages: (i) when they were about 3 months old and mainly had early leaves, (ii) when they were about 6 months old and primarily had intermediate leaves, (iii) when seedlings had intermediate and late leaves and were about 9 months old, and (iv) when they mainly had late leaves and were about 12 months. The seedlings were grown under the conditions described above, and for each stage, we sampled leaves from 8 to 10 seedlings (cone-tainers). Leaves were harvested from the upper half of the seedlings to avoid using old or senescing leaves. Leaf area and SLA were determined by measuring the combined leaf area and dry weight of about 30–40 leaves per seedling, except for the 9-month-old seedlings. For these seedlings, we harvested 30–40 intermediate leaves and a similar number of late leaves. Photos of the leaves were used to measure their area using the ImageJ software (Rueden et al. 2017). Then, the leaves were dried in an oven at 105°C until no changes in weight occurred between successive days. The leaf area-to-weight ratio gave the SLA value (Lambers and Oliveira 2019).

Before sampling seedlings for π_0_ measurements, we watered the cone-tainers to saturation and enclosed the shoots overnight in a dark plastic bag to bring the leaves to near full hydration. This condition was ascertained by measuring the Ψ_l_, which ranged between −0.15 and −0.25 MPa. π_o_ was determined by vapour pressure osmometry of expressed sap (see below for more details). In preliminary work, we also tested the freeze-thawed leaf disc method described by Bartlett et al. (2012a) for determining π_0_. The differences between the two methods were within 0.1 MPa and not statistically significant for similar samples. However, due to the narrowness of *A. tridentata* leaves, the leaf disc method required using three to four leaf fragments to cover the sample holder of the osmometer (VAPRO 5520 osmometer, Wescor, Logan, UT, USA), and, for late leaves, equilibration times after puncturing them 10–15 times were typically longer than 45 minutes, which is much longer than the 80 seconds equilibration with expressed sap. Due to the latter's convenience, the values reported in this study were all obtained using expressed sap. To extract the sap, we placed 50–70 mg of leaves in aluminum foil and submerged them in liquid N for at least 1 hour. The leaves were then transferred to a microcentrifuge tube filter (Spin-X Costar), macerated with a small rod, and centrifuged at 14 000 *g* for 15 minutes. We calibrated the osmometer each day of measurement and then determined the osmotic potentials of the filtrates obtained after centrifugation. Osmometer measurements were made as recommended by the manufacturer.

Due to the heteroscedasticity of the data, differences in leaf area, SLA, and π_0_ between early, intermediate, and late leaves were assessed using Welch's ANOVA and the Games–Howell *post hoc* test as implemented in the Pingouin library in Python (Vallat 2018). The normality of residuals was ascertained by the Shapiro–Wilk test. From the π_0_ values, we also estimated the turgor loss point (π_tlp_) using the regression equation developed by Bartlett et al. (2012a): π_tlp_ = 0.832 × π_0_ − 0.63 (Equation 1). This equation is considered a good predictor of the π_tlp_ estimated from pressure-volume curves in leaves with a wide range of structural and drought tolerance characteristics (Bartlett et al. 2012b, Maréchaux et al. 2016, Kunert et al. 2021).

### Transpiration and photosynthetic responses to water stress in early and late leaves

Transpiration and photosynthesis responses to water stress were characterized at two stages of seedling development: when the seedlings primarily had early leaves and were ∼3 months old, and when they primarily had late leaves and were ∼12 months old. Water stress was imposed by withholding watering, and two approaches were used to determine the sensitivity of the leaves to this stress. As a first approach to determine if seedlings with distinct leaf types responded differently to drought, we measured changes in whole-shoot transpiration by weight and analysed the Ψ_l_ when the transpiration rate reached ∼20% of the maximum observed; reaching this rate at a higher Ψ_l_ would indicate higher stomatal sensitivity to Ψ_l_ (Klein 2014). The second approach involved measuring the transpiration per unit leaf area (Tr), net photosynthesis (NP), stomatal conductance (g_s_), and photosystem II operating efficiency (ΦPSII) at Ψ_l_ values ranging from −0.25 to −4.0 MPa. Differences in the transpiration and photosynthetic decline between early and late leaves in response to decreases in Ψ_l_ would also indicate differences in their ability to function as water stress develops.

To analyse whole-shoot transpiration, the cone-tainers of well-watered seedlings were double-bagged to minimize soil evaporation (Rodriguez-Dominguez and Brodribb 2020). Subsequently, each cone-tainer (containing one seedling) was placed on a digital scale built using an HX load cell amplifier kit (WWZMDiB, 1 kg) connected to a Raspberry Pi Pico W (Supplementary Figure S1). A total of 18 seedlings were evaluated, comprising nine with early leaves and nine with late ones. During exposure to stress, plants were grown under a 14-hour photoperiod with day/night conditions of 23/21 ± 2°C and 20/23 ± 4% relative humidity. LED lights provided 500 µmol m^−2^ s^−1^ of PAR, and fans moved air throughout the seedlings to keep uniform conditions around the plants. Weight measurements were recorded every 30 minutes and uploaded to the Blynk cloud platform (https://blynk.io/). We calculated the average hourly transpiration for each day since withholding water from the weight changes measured between 8 a.m. and 2 p.m. (part of the light period). Supplementary Figure S1 illustrates representative traces of weight and transpiration changes. When transpiration rates reached ∼20% of the highest measured value, the shoot was enclosed in a plastic bag, excised, and immediately used to measure its Ψ_l_ with a pressure chamber (PMS Instrument Company; Albany, OR, USA).

To impose water stress on seedlings used for photosynthesis measurements, seedlings were withheld from watering under the conditions described above. We also estimated changes in transpiration using the scales as a coarse approach to monitor water stress. Seedlings (cone-tainers) were used for gas exchange analyses after undergoing transpiration reductions ranging from 0% to 80%. Net photosynthesis (NP), transpiration (Tr), stomatal conductance (g_s_), and photosystem II operating efficiency (ΦPSII) were measured using a LI-6400-40 leaf chamber fluorometer connected to a LI-COR LI-6400XT portable photosynthesis system (LI-COR Inc., Lincoln, NE, USA). The upper portion of the shoot was used for the measurements, mainly exposing relatively young, fully expanded leaves. Due to the clustered arrangement and small leaf size, there was some overlap between the leaves in the chamber. Leaves were spread, and a few were removed to reduce overlapping (Reed and Loik 2016). Also, the leaves did not always cover the entire leaf chamber, which was circular with an area of 1.77 cm^2^. In these cases, after placing the leaves in the chamber and before closing it, we took pictures of the leaves within the chamber. From these digital images and using the diameter of the chamber for calibration, we determined the leaf area using ImageJ and used it to correct the measured gas exchange parameters (Reed and Loik 2016). NP, Tr, and g_s_ were determined at an incoming airflow of about 200 µmol s^−1^, a CO_2_ concentration of 400 µmol mol^−1^, 25°C, and 1000 µmol m^−2^ s^−1^ light intensity. Values of NP, Tr, and g_s_ were recorded after the CO_2_ assimilation rates and stomatal conductance values had become stable, and the infrared gas analyzer was matched before each measurement. After the gas exchange measurements were completed and to provide supplementary information on photosynthesis, ΦPSII was determined in the same leaves by measuring the steady-state fluorescence (F′) and the maximal fluorescence (Fm’). The latter was measured following a light-saturating pulse of 8000 µmol m^−2^ s^−1^. We also used the NP and g_s_ data to calculate the intrinsic water use efficiency (iWUE), which is the NP/g_s_ ratio. After completing the gas exchange and chlorophyll fluorescence measurements, the shoot portion within the leaf chamber was wrapped with Parafilm, excised, and immediately used to measure its Ψ_l_ with a pressure chamber. Due to the relatively small size of the seedlings and the partially destructive nature of the Ψ_l_ measurement, we no longer used the seedlings after the Ψ_l_ measurements. Thus, the relationship between Ψ_l_ and other parameters was established from measurements conducted in different seedlings.

Differences between early and late leaves in Ψ_l_ at ∼20% of maximum whole shoot transpiration were examined by *t*-test. The effects of Ψ_l_, leaf type, and their interaction on Tr, g_s_, and NP were analysed using a generalized linear mixed model (GLM) with a gamma distribution and a log link function, employing the sm.GLM function in the Statsmodels package in Python 3.10 (Seabold and Perktold 2010). The log link function and gamma distribution enabled us to model the lack of linearity in the relationship between Ψ_l_ and the dependent variables, as well as the increase in residual errors at high water potentials (Bolker 2015). Low deviance values were used to ascertain the fitness of this model.

The decrease in g_s_ with declining Ψ_l_ was also modelled using a sigmoidal function: g_s_ = g_s_max/(1 + (Ψ_l_/Ψg_s_50)^s^_)_ (Equation 2), where g_s_max is the maximal stomatal conductance, Ψg_s_50 the leaf water potential at 50% of g_s_max, and *s* a parameter that affects the shape of the curve (Guyot et al. 2012, Klein 2014). The data for each leaf type were fitted to equation (2) to obtain the values for g_s_max, Ψg_s_50, and *s*. For this purpose, we utilized the Non-Linear Least-Square Minimization and Curve-Fitting library (LMFIT) in Python (Newville et al. 2016). We used a similar approach to estimate *s* for NP and Tr, as well as the maximum NP (NPmax), maximum Tr (Trmax), and the Ψ_l_ at 50% of these values, ΨNP50 and ΨTr50. Differences in maxima, Ψ50, and *s* for g_s_, NP, and Tr between early and late leaves were evaluated by the 95% confidence interval for the difference between means.

The values of NPmax estimated with the sigmoidal model represent NPmax per unit area (µmol CO_2_ m^−2^ s^−1^). These values were multiplied by the average SLA values of early and late leaves (expressed as m^2^ g^−1^) to infer their NPmax per unit mass (µmol CO_2_ g^−1^ s^−1^). In addition, we estimated the 95% CI for these products using conventional formulas for propagation of errors.

### Effects of water stress on leaf area, SLA, and π_0_ after return to well-watered conditions

For this experiment, seedlings were grown under well-watered conditions until they were 2 or 4 months old; at these times, they had mainly early leaves. When the seedlings were 2 months old, a subset of the seedlings was randomly selected and exposed to water stress. For this purpose, we doubled-bagged the containers to minimize soil evaporation and stopped watering until whole-plant transpiration declined to an average of ∼10% of the initial value. When canopy transpiration reached the targeted value of 10% of the initial, we sub-irrigated the cone-tainers once and repeated this drying and watering procedure twice. Each drying cycle lasted about 10 days, and daily changes in transpiration were determined by weight differences. At the end of the third drying cycle, eight seedlings were sampled to estimate the Ψ_l_ of the seedlings at this time. After these measurements, seedlings in the other cone-tainers were sub-irrigated daily for 1 or 3 months to allow new leaf development under well-watered conditions. We sampled seedlings 1 and 3 months after initiating rewatering to determine leaf area, SLA, and π_0_. A similar procedure was carried out when the seedlings were 4 months old, but sampling for analysis of leaf size, SLA, and π_0_ was only conducted after 3 months of rewatering. In addition, seedlings that were kept continuously well-watered were harvested for similar analyses when they were 4 months old. In summary, seedlings were harvested for measurements of leaf area, SLA, and π_0_ after experiencing one of the following watering treatments: 4 months of continuous well-watering (W4), 2 months well-watered, drought-stressed for 1 month, and then rewatered for either 1 month (W2-D1-R1) or 3 months (W2-D1-R3), and 4 months well-watered, drought-stressed for 1 month, and rewatered for 3 months (W4-D1-R3). In all cases, drought-stressed for 1 month refers to the three cycles of drying and rewatering noted above. For each treatment, we harvested 20 seedlings; 12 were used to determine π_0_, and the remaining 8 were used to estimate leaf area and SLA, following the procedures described earlier.

Differences in leaf size between treatments were evaluated by one-way ANOVA and Tukey HSD post-hoc test using the Statsmodels package in Python (Seabold and Perktold 2010). To account for heteroscedasticity in the SLA and π_0_ data, we assessed differences between treatments using Welch's ANOVA and the Games–Howell *post hoc* test, as implemented in the Pingouin library (Vallat 2018). For the three variables, the normality of residuals was ascertained using the Shapiro–Wilk test.

## Results

### Leaf area, SLA, and π_0_ of early, intermediate, and late leaves

**Figure 2.**
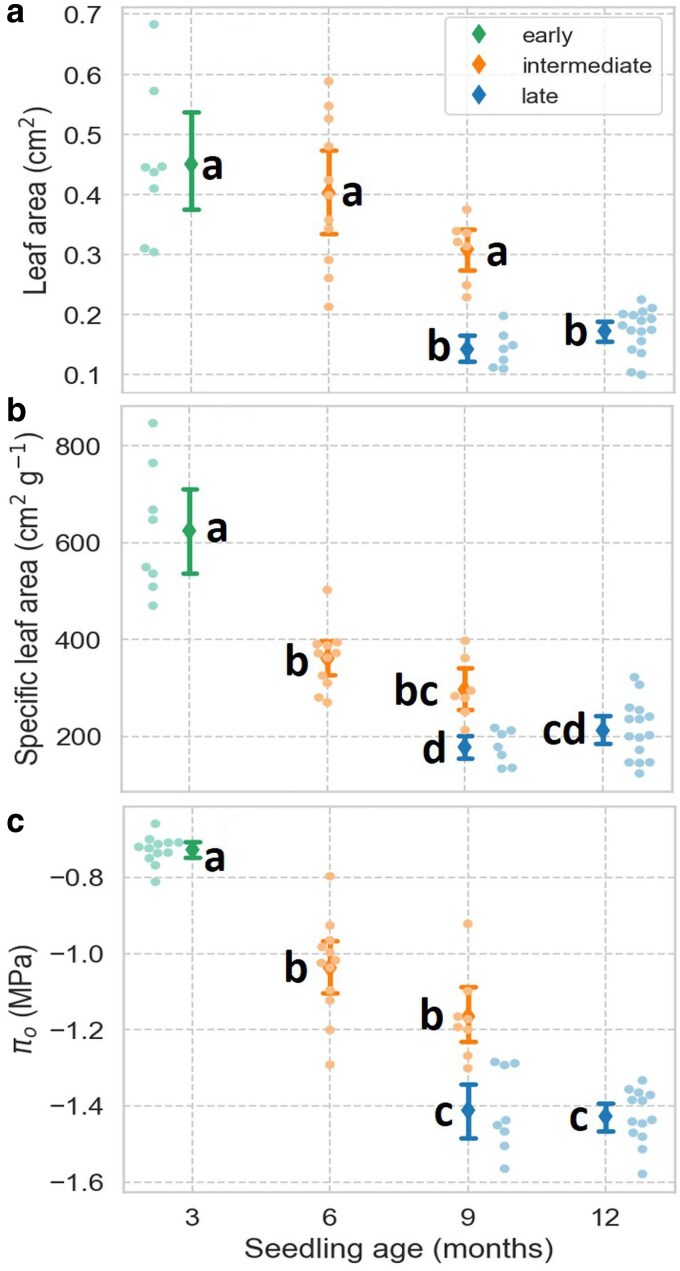
Leaf area (a), specific leaf area (b), and osmotic potential at full turgor (π_0_, c) in *Artemisia tridentata* seedlings at different developmental stages. Means ± 95% CI; means not labelled by the same letter are significantly different (*P* < 0.05).

Early and intermediate leaves showed greater variability in leaf area than late leaves, and there were no significant differences between early and intermediate leaves (Fig. 2a). On average, early and intermediate leaves were 2.8 and 2.2 times larger than late leaves, respectively (*P* < 0.004). SLA also decreased from early to late leaves; however, in contrast to leaf area, the SLA of early leaves was higher than that of intermediate leaves (*P* < 0.004). There was also a decrease in SLA between the leaves present in 6-month-old seedlings and the late leaves of 9 and 12-month-old plants (*P* = 6.1 × 10^−5^) (Fig. 2b). For π_0_, the average values differed between the three types of leaves (Fig. 2c), with an overall decline in π_0_ from about −0.72 MPa in early leaves to −1.44 MPa in late leaves (*P* = 6.3 × 10^−7^). Using Equation 1, average values and 95% CI for the π_tlp_ were −1.23 (±0.03), −1.55 (±0.05), and −1.82 (±0.04) MPa for the early, intermediate, and late leaves, respectively.

### Transpiration and photosynthetic responses to water stress in early and late leaves

**Figure 3.**
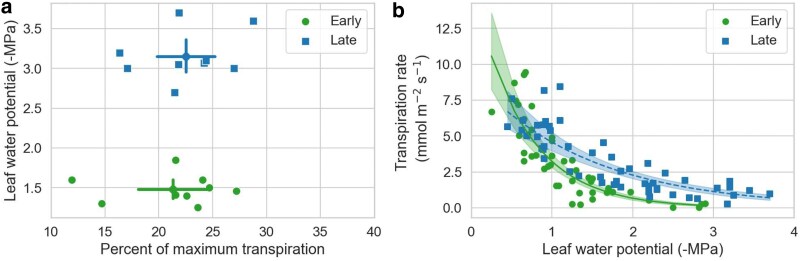
Differences in transpiration between early leaves in 3-month-old seedlings and late leaves in 12-month-old seedlings of *Artemisia tridentata*. (a) Leaf water potential at about 20% of whole-shoot maximum transpiration; diamonds and error bars indicate means and 95% CI. (b) Leaf transpiration rates in response to decreases in leaf water potential; data fitted to a generalized linear model with a gamma distribution and a log-link function; each line indicates the best fit and its 95% CI.

After withholding watering and letting the seedlings reach whole-shoot transpiration rates of around 20% of maximal transpiration, the Ψ_l_ of seedlings with early leaves were, on average, 1.67 MPa higher than those with late leaves (Fig. 3a, *P* < 0.0001). This ability of the late leaves to maintain transpiration to lower Ψ_l_ was consistent with the results obtained at the leaf level using the Li-Cor photosynthesis system (Fig. 3b). The GLM model indicated a significant effect of Ψ_l_ (*P* < 0.0001) and a significant interaction between Ψ_l_ and leaf type (*P* < 0.0001) on the leaf transpiration rate (Tr). In particular, the decline in Tr with Ψ_l_ was steeper in early than late leaves (Fig. 3b).

**Figure 4.**
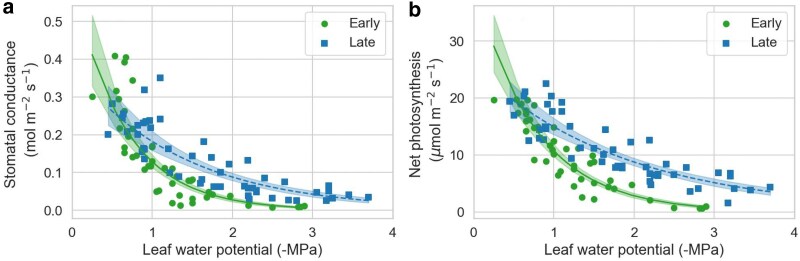
Stomatal conductance (a) and net photosynthesis (b) in response to decreasing leaf water potentials (ψ_l_) for early leaves in 3-month-old seedlings and late leaves in 12-month-old seedlings of *Artemisia tridentata*. Data fitted to a generalized linear model with a gamma distribution and a log-link function; each line indicates the best fit and its 95% CI.

Stomatal conductance (g_s_) and net photosynthesis (NP) exhibited comparable trends to those of Tr. The decline in g_s_ and NP with decreasing Ψ_l_ was more gradual in late than in early leaves (Fig. 4). As a result, late leaves showed some positive net CO_2_ assimilation down to values of −3 MPa, which was not the case for early leaves. These differences in sensitivity to decreases in Ψ_l_ between early and late leaves were significant, as indicated by the interaction term between Ψ_l_ and leaf type, which had *P* values below 0.0001 for both gs and NP.

We also analysed the Tr, g_s_, and NP data using a sigmoidal model (Equation 2) to estimate values for Trmax, g_s_max, and NPmax, as well as the water potentials at half these values (Ψ50) (Supplementary Figure S2). The Trmax, g_s_max, and NPmax values predicted by the sigmoidal model for early leaves, albeit numerically higher, were not significantly different from those of late leaves (Table 1). In contrast, values for ΨTr50, Ψg_s_50, and ΨNP50 were higher in early than late leaves by between 0.6 and 0.77 MPa (Table 1, Supplementary Figure S2). The NPmax values estimated by the sigmoidal model were also used to infer the NPmax per unit mass by multiplying the former by the SLA values of early and late leaves shown in Fig. 2. This estimation yielded a mean and 95% CI of 1.36 (±0.42) µmol CO_2_ g^−1^ s^−1^ for early leaves and of 0.40 (±0.21) µmol CO_2_ g^−1^ s^−1^ for late leaves, indicating that the NPmax per unit mass was higher in early leaves.

**Table 1.** Values for the parameters estimated using the sigmoidal model (Equation 2): Maximum values, leaf water potential at half the maximum values, and s for transpiration rate (Tr), stomatal conductance (g_s_), and net photosynthesis (NP) for early and late leaves present in 3- and 12-month-old *Artemisia tridentata* seedlings, respectively.

Parameter	Early	Late	95% CI of the mean difference
Trmax (mmol m^−2^ s^−1^)	8.02 (−1.70/+2.67)	6.63 (−1.18/+3.03)	(−1.37, 4.14)
ΨTr50 (MPa)	−0.90 (−0.18/+0.19)	−1.50 (−0.34/+0.55)	(0.12, 1.086)^a^
*s*Tr	3.49 (−1.19/+2.07)	2.67 (−1.06/+1.61)	(−1.06, 2.71)
g_s_max (mol m^−2^ s^−1^)	0.36 (−0.08/+0.11)	0.26 (−0.04/+0.09)	(−0.01, 0.21)
Ψg_s_50 (MPa)	−0.84 (−0.15/+0.16)	−1.54 (−0.31/+0.47)	(0.26, 1.11)^a^
*s*g_s_	3.76 (−1.23/+2.17)	2.88 (−1.12/+1.74)	(−1.11, 2.87)
NPmax (µmol m^−2^ s^−1^)	21.82 (−3.55/+5.45)	19.94 (−3.05/+7.87)	(−14.37, 18.12)
ΨNP50 (MPa)	−1.01 (−0.18/+0.21)	−1.768 (−0.38/+0.67)	(0.20, 1.33)^a^
*s*NP	2.66 (−0.71/+1.01)	2.16 (−0.83/+1.07)	(−2.06, 3.06)

Means (±95% CI) and 95% CI of the mean difference.

^a^Confidence intervals of the difference that do not contain zero indicate statistical significance (*P* < 0.05).

From the NP and g_s_ values, we also calculated the intrinsic water use efficiency (iWUE). When examined in relation to decreasing Ψ_l_, the iWUE increased faster in early than late leaves (*P* = 0.001, Supplementary Figure S3a). This trend was primarily caused by the faster decline in g_s_ with Ψ_l_ in early leaves than late leaves (Fig. 4a). The increase in iWUE with decreasing g_s_ (*P* < 0.0001) was virtually identical between early and late leaves (*P* = 0.697, Supplementary Figure S3b).

The other photosynthetic parameter measured, ΦPSII, showed a more linear and gradual decline with Ψ_l_ than the gas exchange parameters (Supplementary Figure S4). Still, there was a significant interaction between leaf type and water potential (*P* = 0.003); the average reduction in ΦPSII in early leaves was about 0.045 units per MPa decrease in Ψ_l_, while that of late leaves was less at 0.017 units per MPa decrease.

### Effects of water stress on leaf area, SLA, and π_0_ after return to well-watered conditions

**Figure 5.**
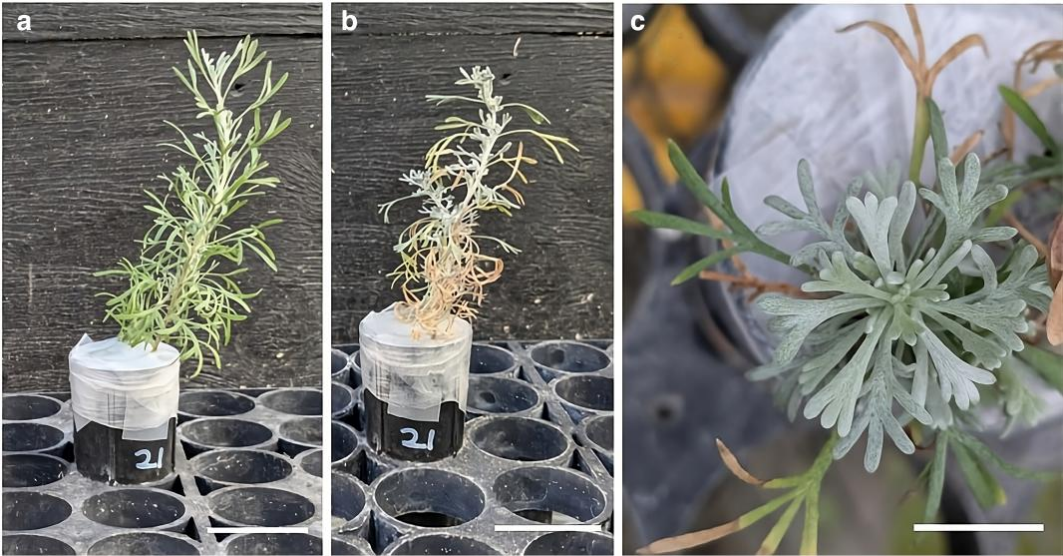
Representative examples of changes in leaf morphology caused by the water stress treatment applied to *Artemisia tridentata* seedlings. (a and b) Show the same plant before and towards the end of the water stress treatment; the plant was 4 months old at the initiation of the stress. c shows changes in leaf morphology in a three-and-a-half-month-old seedling; stress was initiated when the seedling was 2 months old, and the seedlings were rewatered at 3 months. Bars = 38 mm (a, b), 10 mm (c).

Measurements of Ψ_l_ at the end of the third drying cycle showed that the average Ψ_l_ of the stressed plants was 2 MPa lower than that of those well-watered, −0.36 and −2.36 MPa for well-watered and drought-stressed plants, respectively (*P* = 0.002, Supplementary Figure S5). The water stress imposed caused senescence and abscission of most early leaves and the emergence of smaller, more pubescent leaves, which appeared more similar to late leaves (Fig. 5a–c).

**Figure 6.**
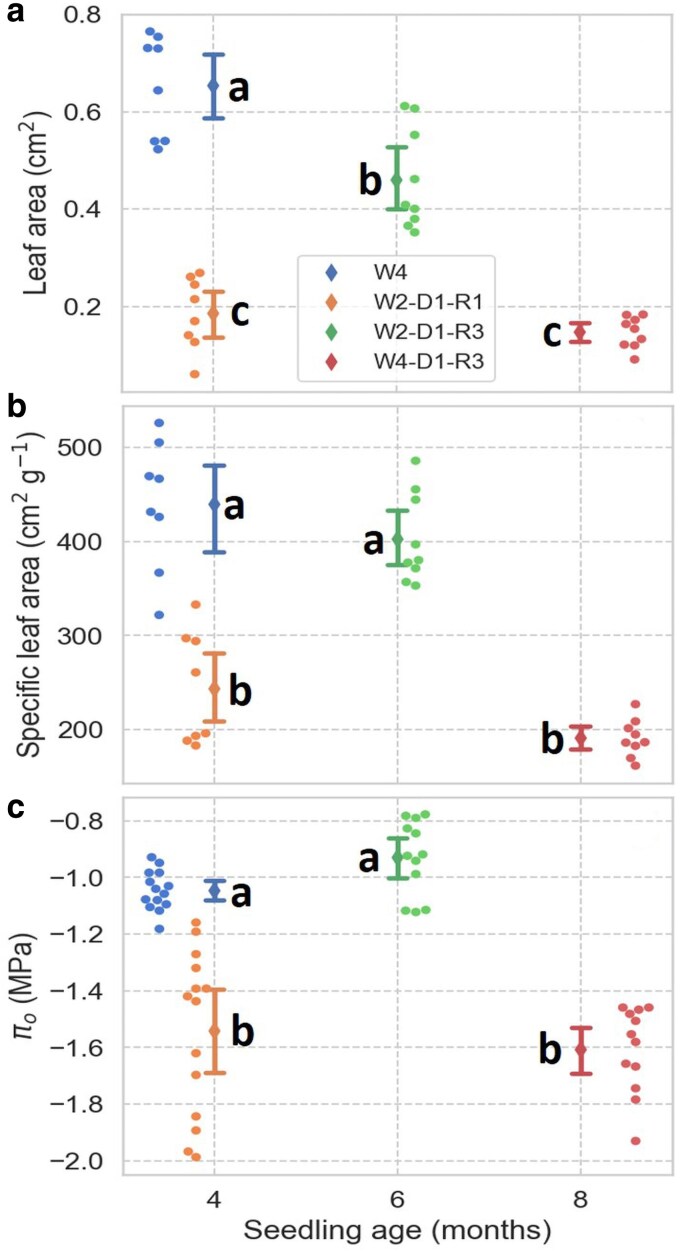
Effect of water stress and rewatering on leaf area (a), specific leaf area (b), and osmotic potential at full turgor (π_0_) (c) of *Artemisia tridentata* seedlings. Seedlings were harvested after exposure to the following treatments: 4 months of continuous well-watered conditions (W4), 2 months well-watered, drought-stressed for 1 month, and rewatered for 1 month (W2-D1-R1), 2 months well-watered, drought-stressed for 1 month, and rewatered for 3 months (W2-D1-R3), and 4 months well-watered, drought-stressed for 1 month, and rewatered for 3 months (W4-D1-R3).

Upon return to constant well-watered conditions, seedlings that experienced drought when they were between 2 and 3 months old continued to form late-like leaves for at least a month. Comparison of these leaves (W2-D1-R1) with those of leaves of seedlings of similar age (∼4 months) but always kept well-watered (W4) indicated differences in size, SLA, and π_0_ (Fig. 6). The average leaf area and SLA in the W2-D1-R1 treatment were about a third and a half of those of the seedlings always well-watered. Similarly, π_0_ in the W2-D1-R1 treatment was 0.5 MPa lower than in the W4 treatment. However, watering for another 2 months (W2-D1-R3) resulted in the development of larger leaves with SLA and π_0_ values similar to those of plants that were always well-watered. Like seedlings stressed when they were 2 months old, seedlings stressed when they were 4 months old dropped most of their early leaves and began forming late-like leaves (Fig. 5a and b). After returning and maintaining these seedlings under well-watered conditions for 3 months (W4-D1-R3 treatment), the newly formed leaves remained small in size, with SLA and π_0_ values similar to those in the W2-D1-R1 treatment (Fig. 6).

## Discussion

The results of this study on seedlings of *A. tridentata* indicate that the observed changes in leaf morphology from early to late leaves were associated with decreases in SLA and π_0_ and an increased capacity to maintain stomatal conductance and photosynthesis at lower water potentials (Figs. 2–4). These results support our first hypothesis that the shifts in leaf morphology during seedling development are associated with increased leaf drought tolerance. Because the changes in leaf functional traits and drought tolerance occurred even when the seedlings were kept well-watered (Fig. 2), the transition from early to late leaves was at least partially caused by ontogenic processes. Additionally, *A. tridentata* seedlings exhibited leaf plasticity in response to changes in water status. Drought triggered the emergence of late-like leaves, and the formation of these leaves continued upon return to well-watered conditions (Fig. 6). These results support our second hypothesis that exposure to drought induces and has a lasting effect on the development of late-like leaves. However, the extent of this response varied depending on the stage of seedling development when the drought occurred. In 3-month-old seedlings, a prolonged period of continuous well-watered conditions led to a return to early-like leaves. In contrast, this was not observed in 5-month-old seedlings, showing less plasticity in leaf development in the latter.

### Differences in functional traits and sensitivity to drought between early and late leaves

The observed decrease in SLA from early to late leaves is comparable to results in other species that show declines in SLA with seedling age or between seedlings and juvenile or adult plants (Niklas and Cobb 2010, Mediavilla et al. 2014, Henn and Damschen 2021). Decreases in SLA and leaf area are thought to signify a switch in allocation of resources (Dayrell et al. 2018). Higher SLA and leaf area tend to favour biomass accumulation and growth (Wright et al. 2004, Dayrell et al. 2018). In contrast, reductions in their values indicate a shift towards a more conservative strategy (Mason et al. 2013, Henn and Damschen 2021). Part of the effect of higher SLA in enhancing growth is that higher SLAs tend to increase net photosynthesis per unit mass (Reich et al. 1998). The results in *A. tridentata* seedlings support this notion. The estimated NP per unit mass was 3-fold higher in early leaves than in late leaves, which corresponded with their differences in SLA. Other things equal, such differences in NPmax per mass would drive faster biomass accumulation and growth in seedlings with early leaves than in those with late ones (Falster et al. 2018).

Like SLA, π_0_ values decreased with seedling age, and the π_tlp_ estimated from π_0_ was 0.59 MPa higher in early than late leaves. This difference largely accounted for the differences in Ψg_s_50 and ΨNP50 between early and late leaves, which were 0.7 and 0.75 MPa, respectively. These results indicate that the ability to maintain turgor due to a higher solute concentration was a significant factor in allowing late leaves to continue photosynthesis at lower water potentials. The effects of π_tlp_ on a plant's ability to cope with drought are complex (Farrell et al. 2017). Species with high π_tlp_ or those that close their stomata above the π_tlp_ reduce transpiration at higher Ψl, which contributes to maintaining high hydration for a longer period (McDowell et al. 2008, Farrell et al. 2017). In some instances, high π_tlp_ has been correlated with increased drought survival; plants with this strategy are referred to as drought avoiders (Sun et al. 2020). In woody plants, however, a more widespread observation is that differences in π_tlp_ among species are negatively correlated with higher survival under drought and with the dryness of the habitat where the species lives (Bartlett et al. 2012b, Maréchaux et al. 2016, Zhu et al. 2018). In these cases, low π_tlp_ values enhance drought tolerance, an effect that has also been reported for seedlings of various woody species (Lazarus et al. 2018, Álvarez-Cansino et al. 2022, Beikircher et al. 2025). Based on these considerations and by broadening the range of Ψ_l_ at which photosynthesis can occur, late leaves would tend to increase the drought tolerance of *A. tridentata* seedlings. As discussed below under ecological implications, maintaining some carbon gain at lower water potential may be crucial for coping with the prolonged summer drought that seedlings experience in nature.

The turgor loss point provides an estimate of the Ψ_l_ below which the stomata are no longer open (Bartlett et al. 2012b, Mitchell and O'Grady 2015). The average π_tlp_ of early and late leaves calculated from the osmometer data was −1.23 and −1.82 MPa, respectively. These values somewhat overestimated the Ψ_l_ at stomatal closure inferred from gas exchange measurements. Using the sigmoidal model (Equation 2) and the values of g_s_max, Ψg_s_50, and *s* shown in Table 1, we estimated the Ψ_l_ at a g_s_ of 0.05 mol m^−2^ s^−1^, which is considered indicative of effective stomatal closure (Farrell et al. 2017). This estimate was −1.37 MPa for early leaves and −2.51 MPa for late leaves, which, primarily for the late leaves, is lower than the π_tlp_ estimated with the osmometer. This discrepancy may reflect errors in the osmometer method, particularly the dilution of cell solutes by apoplastic water, causing less negative values of π_0_ and thereby π_tlp_ (Callister et al. 2006, Bartlett et al. 2012a). Alternatively, if the effect of the apoplastic dilution was minimal, it could indicate other unaccounted factors affecting the π_tlp_, such as cell shrinkage or osmotic adjustment (Evans et al. 1992, Kolb and Sperry 1999). Using the osmometer, we estimated π_tlp_ in seedlings kept under well-watered conditions. Instead, we measured the relationship between Ψ_l_ and g_s_ over a 7− to 10-day drying period, during which drought responses could have contributed to the lower π_tlp_. For adult *A. tridentata* plants, Kolb and Sperry (1999) reported that summer drought lowered π_0_ and π_tlp_ by up to 2 MPa. To a smaller extent, a similar response may have occurred with our seedlings after withholding watering, with the adjustment being more prevalent in late than in early leaves.

In addition to a higher π_tlp_, early leaves showed higher susceptibility to shedding. Within the ranges of Ψ_l_ tested, shedding was minimal for late leaves but extensive for early leaves experiencing Ψ_l_ of about −2 MPa. Shedding at higher Ψ_l_ by early leaves contributes to their more transient nature and reduces water loss, which likely helps maintain the hydraulic integrity of structures with higher carbon investment, such as the stem and late leaves (Hochberg et al. 2017). From a physiological perspective, a remaining question is whether differences in π_tlp_ were related to the differences in leaf shedding. Other studies support such a notion. Drought-induced leaf shedding is often associated with a significant loss in leaf hydraulic conductance (Brodribb and Holbrook 2003, Hochberg et al. 2017, Li et al. 2020). We did not measure leaf hydraulic conductance. However, studies across a range of species have shown a correlation between π_tlp_ and the Ψ_l_ causing a 50% loss of leaf hydraulic conductance (Blackman et al. 2010, Scoffoni et al. 2012, Nardini and Luglio 2014). Such a correlation suggests that the higher π_tlp_ of early leaves would be associated with a loss of leaf hydraulic conductivity and, thereby, a triggering of their shedding at higher Ψ_l_ than late leaves.

### Causes of heteromorphic leaf development

In addition to early and late leaves, *A. tridentata* also exhibited intermediate forms, suggesting gradual changes in morphological and physiological traits. At least under well-watered conditions, these changes may be related to a vegetative phase transition (Chitwood and Otoni 2017, Zhai et al. 2020, Bai et al. 2024). The specific modifications triggered by juvenile-to-adult phase transitions vary between species (Zotz et al. 2011). Still, at the molecular level and in a broad range of taxa, they are regulated by a conserved mechanism that involves microRNA 156 (*MIR156*) and genes within the *SQUAMOSA PROMOTER BINDING PROTEIN-like* (*SPL*) family of transcription factors (Wang et al. 2011, Poethig and Fouracre 2024). A decrease in *MIR156* leads to an increase in the expression of specific *SPL* genes, which, through downstream processes, promote the transition from juvenile to adult traits (Raihan et al. 2021, Poethig and Fouracre 2024). The expression of the *MICRO156/SPL* pathway in *A. tridentata* seedlings needs further investigation (Melton et al. 2022, 2023). Nevertheless, given its highly conserved nature, the *MICRO156/SPL* module appears to be a good candidate for mediating some of the observed changes in leaf characteristics (Manuela and Xu 2020).

While an age-related component can drive vegetative phase changes, environmental factors can delay or shorten the juvenile stage (Cui et al. 2014, Manuela and Xu 2020, Xu et al. 2021). Within this context, the formation of late-like leaves during water stress and after rewatering could be interpreted as drought accelerating the transition from juvenile to adult stages. This idea remains to be tested, but if this were the case, it would differ from responses in other species where factors that reduce growth, including drought and defoliation, postpone the juvenile to adult transition (Yang et al. 2011, Cui et al. 2014, Manuela and Xu 2020, Xu et al. 2021). An alternative possibility is that the drought-induced change was independent of a phase transition but caused by a direct effect of drought on leaf development, leading to heterophylly (Tardieu et al. 2011, Hura et al. 2022). Under this notion, the continued emergence of late-like leaves after rewatering may have been due to short-term stress transcriptional memory favouring the development of more drought-tolerant leaves (Jacques et al. 2021, Sadhukhan et al. 2022). Furthermore, consistent watering can erase this memory (Sadhukhan et al. 2022, Rahman et al. 2025), which could explain the reappearance of early-like leaves in younger seedlings. In contrast, seedlings exposed to drought at 4 months did not revert to early-like leaf development even after 3 months of well-watered conditions. This outcome may have been the result of stress memory and a transition to the adult phase towards the end of the recovery period.

Heteromorphic leaf development and differences in leaf traits can also be related to the shoot type where the leaves are borne (Titman and Wetmore 1955, Niklas and Cobb 2010, Leigh et al. 2011). Adult *A. tridentata* plants have long and short shoots that produce different assortments of leaves. Large ephemeral leaves develop in long shoots, while short shoots, sprouting at the axils of the large ephemerals, produce ephemeral and persistent leaves (Miller and Shultz 1987). At the seedling stage, a parallel situation can be envisioned where the main axis of the seedling and its lateral branches are akin to the long and short shoots of adult plants, respectively. Under well-watered conditions, the late leaves began to form at the tip of the main stem axis. Additionally, drought-induced late-like leaves appeared at the stem's tip and on lateral shoots. Based on these results, heteromorphic leaf development in the seedlings seems independent of stem type.

The changes in leaf traits reported in this study occurred as the seedlings grew under a relatively uniform and favourable environment or in response to drought. In natural habitats, seedlings experience other climatic conditions, such as low temperatures and higher light intensities (Lazarus et al. 2019), which can also influence leaf characteristics and may lead to changes that alter those reported here. We can only conjecture about the nature of changes caused by other environmental conditions. Yet, for cold, a possibility is that the effect is similar to that we showed for drought in 2-month-old seedlings, where the impact of drought on leaf characteristics was reversible. Under this scenario, cold temperatures would favour the development of smaller leaves with lower π_0_, which would contribute to cold resistance (González-Zurdo et al. 2016, Hajihashemi et al. 2018, Xu et al. 2022). However, as the incidence of freezing decreases and cool temperatures become prevalent, the seedlings would form larger leaves with higher SLA and π_0_. Analyses of leaf development under different temperatures would help to test these notions.

### Ecological implications for seedling establishment

The distinct characteristics of early and late leaves suggest that *A. tridentata* evolved under conditions that favoured an acquisitive strategy during early seedling establishment, followed by a switch to a conservative strategy triggered by ontogenic processes or drought. Because sagebrush seeds germinate in late winter to early spring, this dual strategy appears to be a valuable adaptation to an environment with mesic spring conditions and subsequent summer drought. Larger leaves with high SLA during the spring would allow higher CO_2_ fixation per biomass investment, which may be crucial for the seedlings’ rapid growth (Reich et al. 1998, Falster et al. 2018, Lazarus et al. 2019). In semiarid and arid regions, an acquisitive strategy during seedling establishment can be beneficial for accumulating reserves and developing a larger root system that is better able to cope with drought (Matías et al. 2014, Ramírez-Valiente et al. 2021, Solé-Medina and Ramírez-Valiente 2023). In contrast, late leaves are better suited to continue photosynthesis during dry periods, which could allow seedlings to maintain positive carbon balances and/or continue root growth beyond the drying soil front, thereby maintaining water homeostasis (McDowell et al. 2008, Germino and Reinhardt 2014).

One remaining question is the extent to which heteromorphic leaf development helps seedling establishment under the shifting sagebrush steppe conditions. Sagebrush habitats have been experiencing disturbances, particularly those caused by exotic grass invasion and climate change, which increase competition for water directly or through reductions in snowpack and increases in temperature and drought intensity (Donnelly et al. 2018, Shi et al. 2018). These changes tend to accelerate the onset of drought, thereby shortening the period during which seedlings can maintain their early leaves. A transition to a conservative strategy when the seedlings are smaller and less able to explore moister and deeper soil layers may be one of the reasons for the current low rates of establishment (Boyd and Obradovich 2014, Schlaepfer et al. 2014, Anderson et al. 2021).

From a restoration perspective, a practice that can lead to higher establishment by overcoming the limitations of small seedlings is outplanting (Geisler et al. 2022, 2023, Bailey et al. 2024). Still, survival following outplanting can be low, in part due to summer drought mortality (Clements and Harmon 2019). The controlled water stress used in this study has similarities to a nursery technique known as drought hardening, which aims to produce seedlings with more drought-tolerant characteristics (Franco et al. 2006). The effectiveness of this practice in improving survival in natural environments varies; however, a recent meta-analysis identified factors associated with positive effects (Puértolas et al. 2024). The impact of drought hardening on increasing survival is higher in shrubs than in other growth forms and also when seedlings are outplanted to sites with an aridity index below 0.3 (Puértolas et al. 2024). Such values occur through the drier regions of the sagebrush steppe, suggesting that drought hardening would benefit *A. tridentata* seedlings in these regions. Even with high rates of establishment, outplanting is costly and impractical for larger areas (Dettweiler-Robinson et al. 2013). An alternative approach would be to use seeds from genotypes that exhibit developmental patterns that maximize seedling survival in more arid conditions, such as allocating more photosynthates to roots from early stages (Ramírez-Valiente et al. 2021). Common garden experiments and increased knowledge of the *A. tridentata* genome are likely to facilitate the selection of such genotypes (Chaney et al. 2017, Richardson et al. 2021, Melton et al. 2022).

## Conclusions

The heteromorphic leaves of *A. tridentata* seedlings differed in functional traits that affect growth and drought tolerance. The higher SLA and estimated photosynthetic efficiency per biomass of early leaves indicate a higher capacity for growth than late leaves. In contrast, the late leaves were more drought-tolerant. These leaves exhibited a more gradual decrease in photosynthesis and minimal shedding under water stress, which was related to lower π_0_ and π_tlp_ values compared to early leaves. The results also revealed that the shift from early to late leaves and its corresponding change to a more conservative growth strategy can occur within a few months from germination. These changes were induced by drought but eventually occurred under well-watered conditions, suggesting that a juvenile-to-adult transition triggered the latter. After rewatering, drought-stressed seedlings continued to form, at least temporarily, late-like leaves; questions remain as to whether this reflects stress memory or alterations in the juvenile-to-adult transition (Sadhukhan et al. 2022, Poethig and Fouracre 2024). Given the patterns of soil moisture in sagebrush habitats and the timing of *A. tridentata* germination, the presence of early leaves appears to be an adaptive trait that can enhance growth during periods with high water availability. However, the adaptive value of this trait may be diminishing due to vegetation changes that increase competition for water and climatic shifts that shorten the period when early leaves can remain functional.

## Supplementary Material

plaf051_Supplementary_Data

## Data Availability

The data underlying this article are available in Zenodo https://doi.org/10.5281/zenodo.17079832

## References

[plaf051-B1] Álvarez-Cansino L, Comita LS, Jones FA et al Turgor loss point predicts survival responses to experimental and natural drought in tropical tree seedlings. Ecology 2022;103:e3700. 10.1002/ecy.3700 35352828

[plaf051-B2] Anderson RM, Hoose BW, Anderson VJ et al Influence of seed conglomeration technology and planting season on Wyoming big sagebrush restoration. Rangel Ecol Manag 2021;77:126–35. 10.1016/j.rama.2021.04.004

[plaf051-B3] Applestein C, Caughlin TT, Germino MJ. Weather affects post-fire recovery of sagebrush-steppe communities and model transferability among sites. Ecosphere 2021;12:e03446. 10.1002/ecs2.3446

[plaf051-B4] Aronne G, De Micco V. Seasonal dimorphism in the Mediterranean *Cistus incanus* L. subsp. *incanus*. Ann Bot 2001;87:789–94. 10.1006/anbo.2001.1407

[plaf051-B5] Bai X, Chen Z, Chen M et al Morphological, anatomical, and physiological characteristics of heteroblastic *Acacia melanoxylon* grown under weak light. Plants 2024;13:870. 10.3390/plants13060870 38592868 PMC10974800

[plaf051-B6] Bailey EC, Thacker E, Monaco TA et al Transplanted sagebrush “wildlings” exhibit higher survival than greenhouse-grown tubelings yet both recruit new plants. BMC Ecol Evol 2024;24:50. 10.1186/s12862-024-02236-z 38649814 PMC11034100

[plaf051-B7] Bartlett MK, Scoffoni C, Ardy R et al Rapid determination of comparative drought tolerance traits: using an osmometer to predict turgor loss point. Methods Ecol Evol 2012a;3:880–8. 10.1111/j.2041-210X.2012.00230.x

[plaf051-B8] Bartlett MK, Scoffoni C, Sack L. The determinants of leaf turgor loss point and prediction of drought tolerance of species and biomes: a global meta-analysis. Ecol Lett 2012b;15:393–405. 10.1111/j.1461-0248.2012.01751.x 22435987

[plaf051-B9] Bates JD, Davies KW. Effects of conifer treatments on soil nutrient availability and plant composition in sagebrush steppe. For Ecol Manage 2017;400:631–44. 10.1016/j.foreco.2017.06.033

[plaf051-B10] Beikircher B, Held M, Losso A et al New insights into a sensitive life stage: hydraulics of tree seedlings in their first growing season. New Phytol 2025;245:577–90. 10.1111/nph.20243 39501601 PMC11655438

[plaf051-B11] Blackman CJ, Brodribb TJ, Jordan GJ. Leaf hydraulic vulnerability is related to conduit dimensions and drought resistance across a diverse range of woody angiosperms. New Phytol 2010;188:1113–23. 10.1111/j.1469-8137.2010.03439.x 20738785

[plaf051-B12] Bolker BM . Linear and generalized linear mixed models. In: Fox GA, Negrete-Yankelevich S, Sosa VJ (eds.) Ecological Statistics. Oxford: Oxford University Press, 2015, 309–33.

[plaf051-B13] Boyd CS, Obradovich M. Is pile seeding Wyoming big sagebrush (*Artemisia tridentata* subsp. *wyomingensis*) an effective alternative to broadcast seeding? Rangel Ecol Manag 2014;67:292–7. 10.2111/REM-D-13-00107.1

[plaf051-B14] Brabec MM, Germino MJ, Richardson BA. Climate adaption and post-fire restoration of a foundational perennial in cold desert: insights from intraspecific variation in response to weather. Journal of Applied Ecology 2017;54:293–302. 10.1111/1365-2664.12679

[plaf051-B15] Brodribb TJ, Holbrook NM. Changes in leaf hydraulic conductance during leaf shedding in seasonally dry tropical forest. New Phytol 2003;158:295–303. 10.1046/j.1469-8137.2003.00736.x

[plaf051-B16] Brooks ML, D’Antonio CM, Richardson DM et al Effects of invasive alien plants on fire regimes. Bioscience 2004;54:677–88. 10.1641/0006-3568(2004)054[0677:EOIAPO]2.0.CO;2

[plaf051-B17] Callister AN, Arndt SK, Adams MA. Comparison of four methods for measuring osmotic potential of tree leaves. Physiol Plant 2006;127:383–92. 10.1111/j.1399-3054.2006.00652.x

[plaf051-B18] Chaney L, Richardson BA, Germino MJ. Climate drives adaptive genetic responses associated with survival in big sagebrush (*Artemisia tridentata*). Evol Appl 2017;10:313–22. 10.1111/eva.12440 28352292 PMC5367076

[plaf051-B19] Charley J, West N. Micro-patterns of nitrogen mineralization activity in soils of some shrub-dominated semi-desert ecosystems of Utah. Soil Biol Biochem 1977;9:357–65. 10.1016/0038-0717(77)90010-4

[plaf051-B20] Chen H-C, Hwang S-G, Chen S-M et al ABA-mediated heterophylly is regulated by differential expression of 9-cis-epoxycarotenoid dioxygenase 3 in lilies. Plant Cell Physiol 2011;52:1806–21. 10.1093/pcp/pcr117 21865303

[plaf051-B21] Chitwood DH, Otoni WC. Divergent leaf shapes among *Passiflora* species arise from a shared juvenile morphology. Plant Direct 2017;1:e00028. 10.1002/pld3.28 31245674 PMC6508542

[plaf051-B22] Chitwood DH, Sinha NR. Evolutionary and environmental forces sculpting leaf development. Curr Biol 2016;26:R297–306. 10.1016/j.cub.2016.02.033 27046820

[plaf051-B23] Clements CD, Harmon D. Survivability of Wyoming big sagebrush transplants. Rangelands 2019;41:88–93. 10.1016/j.rala.2018.11.008

[plaf051-B24] Cui L-G, Shan J-X, Shi M et al The miR156-9- pathway coordinates the relationship between development and abiotic stress tolerance in plants. Plant J 2014;80:1108–17. 10.1111/tpj.12712 25345491

[plaf051-B25] Dantonio CM, Vitousek PM. Biological invasions by exotic grasses, the grass fire cycle, and global change. Annu Rev Ecol Syst 1992;23:63–87. 10.1146/annurev.es.23.110192.000431

[plaf051-B26] Davies KW, Bates JD, Miller RF. The influence of *Artemisia tridentata* ssp *wyomingensis* on microsite and herbaceous vegetation heterogeneity. J Arid Environ 2007;69:441–57. 10.1016/j.jaridenv.2006.10.017

[plaf051-B27] Dayrell RLC, Arruda AJ, Pierce S et al Ontogenetic shifts in plant ecological strategies. Funct Ecol 2018;32:2730–41. 10.1111/1365-2435.13221

[plaf051-B28] De Kroon H, Huber H, Stuefer JF et al A modular concept of phenotypic plasticity in plants. New Phytol 2005;166:73–82. 10.1111/j.1469-8137.2004.01310.x 15760352

[plaf051-B29] Dettweiler-Robinson E, Bakker JD, Evans JR et al Outplanting Wyoming big sagebrush following wildfire: stock performance and economics. Rangel Ecol Manag 2013;66:657–66. 10.2111/REM-D-12-00114.1

[plaf051-B30] DiCristina K, Germino M. Correlation of neighborhood relationships, carbon assimilation, and water status of sagebrush seedlings establishing after fire. West N Am Nat 2006;66:441–9. 10.3398/1527-0904(2006)66[441:CONRCA]2.0.CO;2

[plaf051-B31] Doherty KE, Maestas J, Remington T et al State of the Sagebrush: implementing the sagebrush conservation design to save a biome. Rangel Ecol Manag 2024;97:1–11. 10.1016/j.rama.2024.08.017

[plaf051-B32] Donnelly JP, Allred BW, Perret D et al Seasonal drought in North America’s sagebrush biome structures dynamic mesic resources for sage-grouse. Ecol Evol 2018;8:12492–505. 10.1002/ece3.4614 30619560 PMC6308899

[plaf051-B33] Evans RD, Black RA, Loescher WH et al Osmotic relations of the drought-tolerant shrub *Artemisia tridentata* in response to water stress. Plant Cell Environ 1992;15:49–59. 10.1111/j.1365-3040.1992.tb01457.x

[plaf051-B34] Falster DS, Duursma RA, FitzJohn RG. How functional traits influence plant growth and shade tolerance across the life cycle. Proc Natl Acad Sci 2018;115:E6789–98. 10.1073/pnas.1714044115 29959205 PMC6055161

[plaf051-B35] Farrell C, Szota C, Arndt SK. Does the turgor loss point characterize drought response in dryland plants? Plant Cell Environ 2017;40:1500–11. 10.1111/pce.12948 28342210

[plaf051-B36] Franco JA, Martinez-Sanchez JJ, Fernandez JA et al Selection and nursery production of ornamental plants for landscaping and xerogardening in semi-arid environments. J Horticult Sci Biotechnol 2006;81:3–17. 10.1080/14620316.2006.11512022

[plaf051-B37] Geisler M, Buerki S, Serpe MD. Herbivory amplifies adverse effects of drought on seedling recruitment in a keystone species of western North American rangelands. Plants 2022;11:2628. 10.3390/plants11192628 36235494 PMC9573362

[plaf051-B38] Geisler M, Buerki S, Serpe MD. Arbuscular mycorrhizae alter photosynthetic responses to drought in seedlings of *Artemisia tridentata*. Plants 2023;12:2990. 10.3390/plants12162990 37631200 PMC10458374

[plaf051-B39] Germino MJ, Reinhardt K. Desert shrub responses to experimental modification of precipitation seasonality and soil depth: relationship to the two-layer hypothesis and ecohydrological niche (R Jones, Ed.). J Ecol 2014;102:989–97. 10.1111/1365-2745.12266

[plaf051-B40] González-Zurdo P, Escudero A, Babiano J et al Costs of leaf reinforcement in response to winter cold in evergreen species. Tree Physiol 2016;36:273–86. 10.1093/treephys/tpv134 26764268 PMC4885944

[plaf051-B41] Guillemot J, Martin-StPaul NK, Bulascoschi L et al Small and slow is safe: on the drought tolerance of tropical tree species. Glob Chang Biol 2022;28:2622–38. 10.1111/gcb.16082 35007364

[plaf051-B42] Guyot G, Scoffoni C, Sack L. Combined impacts of irradiance and dehydration on leaf hydraulic conductance: insights into vulnerability and stomatal control. Plant Cell Environ 2012;35:857–71. 10.1111/j.1365-3040.2011.02458.x 22070647

[plaf051-B43] Hajihashemi S, Noedoost F, Geuns JMC et al Effect of cold stress on photosynthetic traits, carbohydrates, morphology, and anatomy in nine cultivars of *Stevia rebaudiana*. Front Plant Sci 2018;9:1430. 10.3389/fpls.2018.01430 30323827 PMC6172358

[plaf051-B44] Heil JA, Simler-Williamson A, Striluk ML et al Weather and leaf age separately contribute to temporal shifts in phyllosphere fungal community structure in sagebrush. Ecosphere 2025;16:e70295. 10.1002/ecs2.70295

[plaf051-B45] Henn JJ, Damschen EI. Plant age affects intraspecific variation in functional traits. Plant Ecol 2021;222:669–80. 10.1007/s11258-021-01136-2

[plaf051-B46] Herrera JC, Calderan A, Gambetta GA et al Stomatal responses in grapevine become increasingly more tolerant to low water potentials throughout the growing season. Plant J 2022;109:804–15. 10.1111/tpj.15591 34797611

[plaf051-B47] Hochberg U, Windt CW, Ponomarenko A et al Stomatal closure, basal leaf embolism, and shedding protect the hydraulic integrity of grape stems. Plant Physiol 2017;174:764–75. 10.1104/pp.16.01816 28351909 PMC5462014

[plaf051-B48] Homer CG, Xian G, Aldridge CL et al Forecasting sagebrush ecosystem components and greater sage-grouse habitat for 2050: learning from past climate patterns and Landsat imagery to predict the future. Ecol Indic 2015;55:131–45. 10.1016/j.ecolind.2015.03.002

[plaf051-B49] Hura T, Hura K, Ostrowska A. Drought-stress induced physiological and molecular changes in plants. Int J Mol Sci 2022;23:4698. 10.3390/ijms23094698 35563089 PMC9104437

[plaf051-B50] Jacques C, Salon C, Barnard RL et al Drought stress memory at the plant cycle level: a review. Plants 2021;10:1873. 10.3390/plants10091873 34579406 PMC8466371

[plaf051-B51] Kaproth MA, Fredericksen BW, González-Rodríguez A et al Drought response strategies are coupled with leaf habit in 35 evergreen and deciduous oak (*Quercus*) species across a climatic gradient in the Americas. New Phytol 2023;239:888–904. 10.1111/nph.19019 37282764

[plaf051-B52] Klein T . The variability of stomatal sensitivity to leaf water potential across tree species indicates a continuum between isohydric and anisohydric behaviours. Funct Ecol 2014;28:1313–20. 10.1111/1365-2435.12289

[plaf051-B53] Knick ST, Dobkin DS, Rotenberry JT et al Teetering on the edge or too late? Conservation and research issues for avifauna of sagebrush habitats. Condor 2003;105:611–34. 10.1093/condor/105.4.611

[plaf051-B54] Kolb KJ, Sperry JS. Differences in drought adaptation between subspecies of sagebrush (*Artemisia tridentata*). Ecology 1999;80:2373–84. 10.1890/0012-9658(1999)080[2373:DIDABS]2.0.CO;2

[plaf051-B55] Kunert N, Zailaa J, Herrmann V et al Leaf turgor loss point shapes local and regional distributions of evergreen but not deciduous tropical trees. New Phytol 2021;230:485–96. 10.1111/nph.17187 33449384 PMC8048579

[plaf051-B56] Lambers H, Oliveira RS. Plant Physiological Ecology. Cham: Springer International Publishing, 2019.

[plaf051-B57] Larrucea ES, Brussard PF. Habitat selection and current distribution of the pygmy rabbit in Nevada and California, USA. J Mammal 2008;89:691–9. 10.1644/07-MAMM-A-199R.1

[plaf051-B58] Lazarus BE, Castanha C, Germino MJ et al Growth strategies and threshold responses to water deficit modulate effects of warming on tree seedlings from forest to alpine. J Ecol 2018;106:571–85. 10.1111/1365-2745.12837

[plaf051-B59] Lazarus BE, Germino MJ, Richardson BA. Freezing resistance, safety margins, and survival vary among big sagebrush populations across the western United States. Am J Bot 2019;106:922–34. 10.1002/ajb2.1320 31294835

[plaf051-B60] Leigh A, Zwieniecki MA, Rockwell FE et al Structural and hydraulic correlates of heterophylly in *Ginkgo biloba*. New Phytol 2011;189:459–70. 10.1111/j.1469-8137.2010.03476.x 20880226

[plaf051-B61] Li G, Hu S, Hou H et al Heterophylly: phenotypic plasticity of leaf shape in aquatic and amphibious plants. Plants 2019;8:420. 10.3390/plants8100420 31623228 PMC6843204

[plaf051-B62] Li X, Smith R, Choat B et al Drought resistance of cotton (*Gossypium hirsutum*) is promoted by early stomatal closure and leaf shedding. Funct Plant Biol 2020;47:91–8. 10.1071/FP19093 31825787

[plaf051-B63] Manuela D, Xu M. Juvenile leaves or adult leaves: determinants for vegetative phase change in flowering plants. Int J Mol Sci 2020;21:9753. 10.3390/ijms21249753 33371265 PMC7766579

[plaf051-B64] Maréchaux I, Bartlett MK, Gaucher P et al Causes of variation in leaf-level drought tolerance within an Amazonian forest. J Plant Hydraul 2016;3:e004. 10.20870/jph.2016.e004

[plaf051-B65] Mason CM, McGaughey SE, Donovan LA. Ontogeny strongly and differentially alters leaf economic and other key traits in three diverse *Helianthus* species. J Exp Bot 2013;64:4089–99. 10.1093/jxb/ert249 24078673

[plaf051-B66] Matías L, González-Díaz P, Jump AS. Larger investment in roots in southern range-edge populations of Scots pine is associated with increased growth and seedling resistance to extreme drought in response to simulated climate change. Environ Exp Bot 2014;105:32–8. 10.1016/j.envexpbot.2014.04.003

[plaf051-B67] McDowell N, Pockman WT, Allen CD et al Mechanisms of plant survival and mortality during drought: why do some plants survive while others succumb to drought? New Phytol 2008;178:719–39. 10.1111/j.1469-8137.2008.02436.x 18422905

[plaf051-B68] Mediavilla S, Herranz M, González-Zurdo P et al Ontogenetic transition in leaf traits: a new cost associated with the increase in leaf longevity. J Plant Ecol 2014;7:567–75. 10.1093/jpe/rtt059

[plaf051-B69] Melton AE, Child AW, Beard RS et al A haploid pseudo-chromosome genome assembly for a keystone sagebrush species of western North American rangelands. G3 2022;12:jkac122. 10.1093/g3journal/jkac122 35567476 PMC9258541

[plaf051-B70] Melton AE, Moran K, Martinez P et al A genotype × environment experiment reveals contrasting response strategies to drought between populations of a keystone species (*Artemisia tridentata*; Asteraceae). Plant-Environment Interactions 2023;4:201–14. 10.1002/pei3.10119 37583876 PMC10423975

[plaf051-B71] Miller RF, Shultz LM. Development and longevity of ephemeral and perennial leaves on *Artemisia tridentata* Nutt. ssp. *wyomingensis*. The Great Basin Naturalist 1987;47:227–30.

[plaf051-B72] Mitchell PJ, O’Grady AP. Adaptation of leaf water relations to climatic and habitat water availability. Forests 2015;6:2281–95. 10.3390/f6072281

[plaf051-B73] Mulkey SS, Smith AP, Wright SJ et al Contrasting leaf phenotypes control seasonal variation in water loss in a tropical forest shrub. Proceedings of the National Academy of Sciences 1992;89:9084–8. 10.1073/pnas.89.19.9084 PMC50069 11607329

[plaf051-B74] Nakayama H, Sinha NR, Kimura S. How do plants and phytohormones accomplish heterophylly, leaf phenotypic plasticity, in response to environmental cues. Front Plant Sci 2017;8:1717. 10.3389/fpls.2017.01717 29046687 PMC5632738

[plaf051-B75] Nardini A, Luglio J. Leaf hydraulic capacity and drought vulnerability: possible trade-offs and correlations with climate across three major biomes. Funct Ecol 2014;28:810–8. 10.1111/1365-2435.12246

[plaf051-B76] Newville M, Stensitzki T, Allen DB et al *LMFIT: Non-Linear Least-Square Minimization and Curve-Fitting for Python*. Astrophysics Source Course Library: asci-1616. 2016.

[plaf051-B77] Niklas KJ, Cobb ED. Ontogenetic changes in the numbers of short- vs. long-shoots account for decreasing specific leaf area in *Acer rubrum* (Aceraceae) as trees increase in size. Am J Bot 2010;97:27–37. 10.3732/ajb.0900249 21622364

[plaf051-B78] O’Connor RC, Germino MJ, Barnard DM et al Small-scale water deficits after wildfires create long-lasting ecological impacts. Environ Res Lett 2020;15:044001. 10.1088/1748-9326/ab79e4

[plaf051-B79] Palacio S, Millard P, Montserrat-Martí G. Aboveground biomass allocation patterns within Mediterranean sub-shrubs: a quantitative analysis of seasonal dimorphism. Flora 2006;201:612–22. 10.1016/j.flora.2006.02.002

[plaf051-B80] Poethig RS, Fouracre J. Temporal regulation of vegetative phase change in plants. Dev Cell 2024;59:4–19. 10.1016/j.devcel.2023.11.010 38194910 PMC10783531

[plaf051-B81] Puértolas J, Villar-Salvador P, Andivia E et al Die-hard seedlings. A global meta-analysis on the factors determining the effectiveness of drought hardening on growth and survival of forest plantations. For Ecol Manage 2024;572:122300. 10.1016/j.foreco.2024.122300

[plaf051-B82] Rahman MM, Keya SS, Bulle M et al Past trauma, better future: how stress memory shapes plant adaptation to drought. Funct Plant Biol 2025;52:6. 10.1071/FP24355 40373187

[plaf051-B83] Raihan T, Geneve RL, Perry SE et al The regulation of plant vegetative phase transition and rejuvenation: mirnas, a key regulator. Epigenomes 2021;5:24. 10.3390/epigenomes5040024 34968248 PMC8715473

[plaf051-B84] Ramírez-Valiente JA, Solé-Medina A, Pyhäjärvi T et al Selection patterns on early-life phenotypic traits in *Pinus sylvestris* are associated with precipitation and temperature along a climatic gradient in Europe. New Phytol 2021;229:3009–25. 10.1111/nph.17029 33098590

[plaf051-B85] Reed CC, Loik ME. Water relations and photosynthesis along an elevation gradient for *Artemisia tridentata* during an historic drought. Oecologia 2016;181:65–76. 10.1007/s00442-015-3528-7 26822944

[plaf051-B86] Reich PB, Ellsworth DS, Walters MB. Leaf structure (specific leaf area) modulates photosynthesis–nitrogen relations: evidence from within and across species and functional groups. Funct Ecol 1998;12:948–58. 10.1046/j.1365-2435.1998.00274.x

[plaf051-B87] Richardson BA, Germino MJ, Warwell MV et al The role of genome duplication in big sagebrush growth and fecundity. Am J Bot 2021;108:1405–16. 10.1002/ajb2.1714 34460105

[plaf051-B88] Richardson BA, Page JT, Bajgain P et al Deep sequencing of amplicons reveals widespread intraspecific hybridization and multiple origins of polyploidy in big sagebrush (*Artemisia tridentata*; Asteraceae). Am J Bot 2012;99:1962–75. 10.3732/ajb.1200373 23204489

[plaf051-B89] Rodriguez-Dominguez CM, Brodribb TJ. Declining root water transport drives stomatal closure in olive under moderate water stress. New Phytol 2020;225:126–34. 10.1111/nph.16177 31498457

[plaf051-B90] Rossatto DR, Kolb RM. An evergreen neotropical savanna tree (*Gochnatia polymorpha*, Asteraceae) produces different dry- and wet-season leaf types. Australian Journal of Botany 2009;57:439–43. 10.1071/BT09045

[plaf051-B91] Rueden CT, Schindelin J, Hiner MC et al Imagej2: ImageJ for the next generation of scientific image data. BMC Bioinformatics 2017;18:529. 10.1186/s12859-017-1934-z 29187165 PMC5708080

[plaf051-B92] Sadhukhan A, Prasad SS, Mitra J et al How do plants remember drought? Planta 2022;256:7. 10.1007/s00425-022-03924-0 35687165

[plaf051-B93] Schlaepfer DR, Bradford JB, Lauenroth WK et al Understanding the future of big sagebrush regeneration: challenges of projecting complex ecological processes. Ecosphere 2021;12:e03695. 10.1002/ecs2.3695

[plaf051-B94] Schlaepfer DR, Lauenroth WK, Bradford JB. Natural regeneration processes in big sagebrush (*Artemisia tridentata*). Rangel Ecol Manag 2014;67:344–57. 10.2111/REM-D-13-00079.1

[plaf051-B95] Scoffoni C, McKown AD, Rawls M et al Dynamics of leaf hydraulic conductance with water status: quantification and analysis of species differences under steady state. J Exp Bot 2012;63:643–58. 10.1093/jxb/err270 22016424 PMC3254676

[plaf051-B96] Seabold S, Perktold J. Statsmodels: econometric and statistical modeling with python. In: *Proceedings of the 9th Python in Science Conference, Austin, Texas, ScyPy 7.1*, 2010, 92–6.

[plaf051-B97] Shi H, Rigge M, Homer CG et al Historical cover trends in a sagebrush steppe ecosystem from 1985 to 2013: links with climate, disturbance, and management. Ecosystems 2018;21:913–29. 10.1007/s10021-017-0191-3

[plaf051-B98] Simler-Williamson AB, Germino MJ. Statistical considerations of nonrandom treatment applications reveal region-wide benefits of widespread post-fire restoration action. Nat Commun 2022;13:3472. 10.1038/s41467-022-31102-z 35710763 PMC9203498

[plaf051-B99] Solé-Medina A, Ramírez-Valiente JA. Common garden experiments reveal acquisitive strategies for responding to drought in seedlings of forest tree species: a commentary on ‘Clinal variations in seedling traits and responses to water availability correspond to seed-source environmental gradients in a foundational dryland tree species'. Ann Bot 2023;132:i–ii. 10.1093/aob/mcad115 PMC10583190 37703333

[plaf051-B100] Sun S, Jung E-Y, Gaviria J et al Drought survival is positively associated with high turgor loss points in temperate perennial grassland species. Funct Ecol 2020;34:788–98. 10.1111/1365-2435.13522

[plaf051-B101] Tardieu F, Granier C, Muller B. Water deficit and growth. Coordinating processes without an orchestrator? Curr Opin Plant Biol 2011;14:283–9. 10.1016/j.pbi.2011.02.002 21388861

[plaf051-B102] Titman PW, Wetmore RH. The growth of long and short shoots in *Cercidiphyllum*. Am J Bot 1955;42:364–72. 10.1002/j.1537-2197.1955.tb11133.x

[plaf051-B103] Tripathi S, Bhadouria R, Srivastava P et al Effects of light availability on leaf attributes and seedling growth of four tree species in tropical dry forest. Ecol Process 2020;9:2. 10.1186/s13717-019-0206-4

[plaf051-B104] Vallat R . Pingouin: statistics in Python. J Open Source Softw 2018;3:1026. 10.21105/joss.01026

[plaf051-B105] Vasey GL, Urza AK, Chambers JC et al Clinal variations in seedling traits and responses to water availability correspond to seed-source environmental gradients in a foundational dryland tree species. Ann Bot 2023;132:203–16. 10.1093/aob/mcad041 36905361 PMC10583205

[plaf051-B106] Velazquez-Gonzalez RS, Garcia-Garcia AL, Ventura-Zapata E et al A review on hydroponics and the technologies associated for medium- and small-scale operations. Agriculture 2022;12:646. 10.3390/agriculture12050646

[plaf051-B107] Wang J-W, Park MY, Wang L-J et al MiRNA control of vegetative phase change in trees. PLoS Genet 2011;7:e1002012. 10.1371/journal.pgen.1002012 21383862 PMC3044678

[plaf051-B108] Wells CL, Pigliucci M. Adaptive phenotypic plasticity: the case of heterophylly in aquatic plants. Perspect Plant Ecol Evol Syst 2000;3:1–18. 10.1078/1433-8319-00001

[plaf051-B109] Westman WE . Seasonal dimorphism of foliage in Californian coastal sage scrub. Oecologia 1981;51:385–8. 10.1007/BF00540910 28310024

[plaf051-B110] Winn AA . The functional significance and fitness consequences of heterophylly. Int J Plant Sci 1999;160:S113–21. 10.1086/314222 10572026

[plaf051-B111] Wright IJ, Reich PB, Westoby M et al The worldwide leaf economics spectrum. Nature 2004;428:821–7. 10.1038/nature02403 15103368

[plaf051-B112] Xu M, Hu T, Poethig RS. Low light intensity delays vegetative phase change. Plant Physiol 2021;187:1177–88. 10.1093/plphys/kiab243 34618024 PMC8566249

[plaf051-B113] Xu H, Huang C, Jiang X et al Impact of cold stress on leaf structure, photosynthesis, and metabolites in *Camellia weiningensis* and *C. oleifera* seedlings. Horticulturae 2022;8:494. 10.3390/horticulturae8060494

[plaf051-B114] Yang L, Conway SR, Poethig RS. Vegetative phase change is mediated by a leaf-derived signal that represses the transcription of miR156. Development 2011;138:245–9. 10.1242/dev.058578 21148189 PMC3005601

[plaf051-B115] Zhai JT, Li YL, Han ZJ et al Morphological, structural and physiological differences in heteromorphic leaves of *Euphrates* poplar during development stages and at crown scales. Plant Biology 2020;22:366–75. 10.1111/plb.13078 31793152 PMC7318281

[plaf051-B116] Zhu S-D, He P-C, Li R-H et al Drought tolerance traits predict survival ratio of native tree species planted in a subtropical degraded hilly area in south China. For Ecol Manage 2018;418:41–6. 10.1016/j.foreco.2017.09.016

[plaf051-B117] Zotz G, Wilhelm K, Becker A. Heteroblasty—a review. The Botanical Review 2011;77:109–51. 10.1007/s12229-010-9062-8

