# Supplementary material for: Differences in functional traits and drought tolerance between heteromorphic leaves of Artemisia tridentata seedlings, a keystone species from a semiarid shrubland
Source: AoB Plants. 2025 Sep 14;17(5):plaf051. doi: 10.1093/aobpla/plaf051 (PMC12492002; doi:10.1093/aobpla/plaf051)
Supplement: plaf051_Supplementary_Data [file plaf051_supplementary_data.pdf]

Supplementary Information

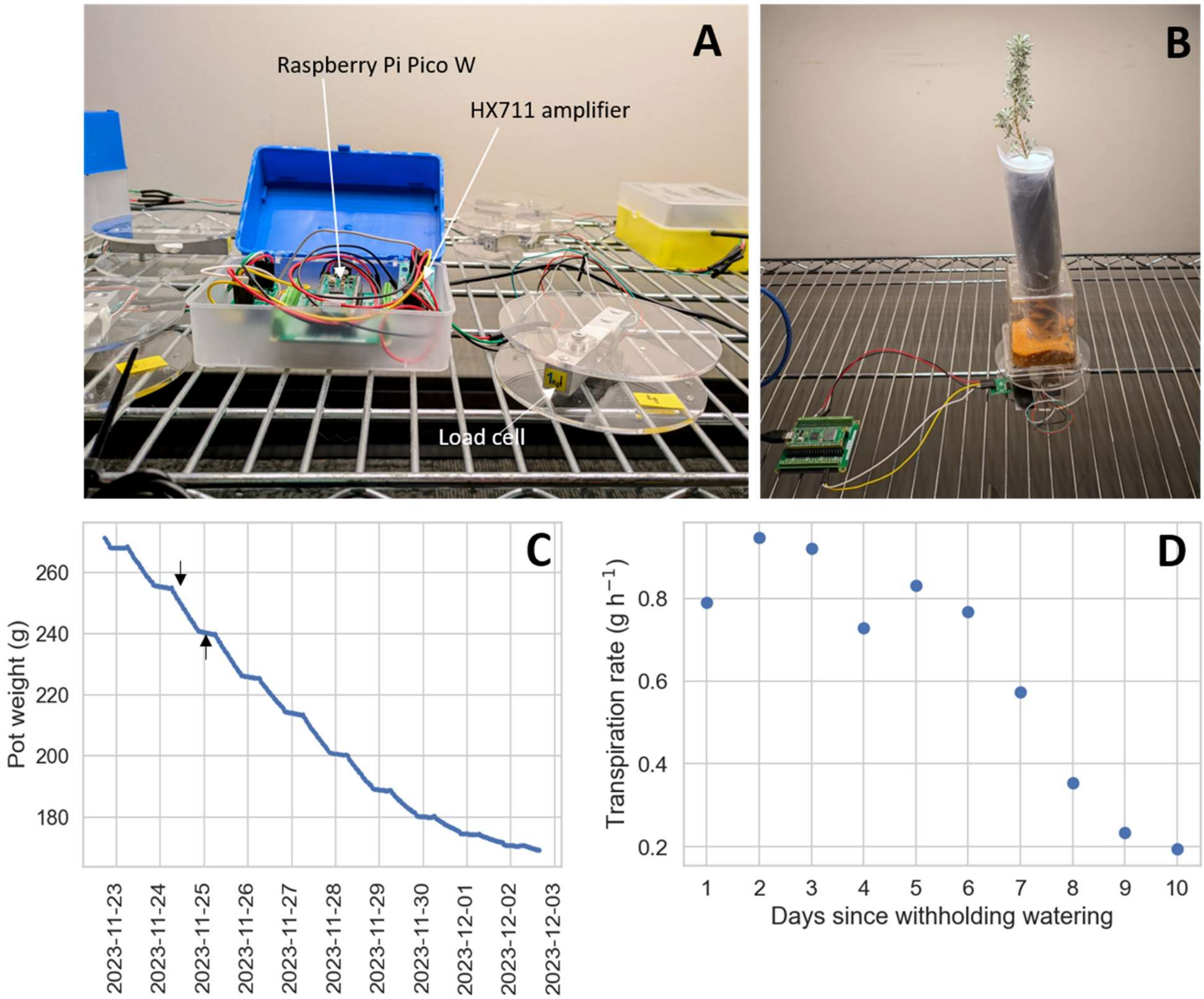

Figure S1. Scales used to measure whole shoot transpiration by weight and representative data. **A,** Each scale consist of a platform with a load cell (the platform supports the weight and the load cell is a transducer that converts weight into an electric signal), an HX711 amplifier that magnifies the signal and converts it to a digital value, and a microcontroller that receives the signal and sends it to the web; in our case the the Raspberry Pi picoW was the microcontroller and Blynk the app that received and stored the weight values. **B,** A scale with the setup used to support the cone-tainers and minimize soil evaporation. **C,** Changes in pot weight after withholding watering; down and up arrows point to the transition from dark to light and light to

dark conditions, respectively. **D,** Average transpiration rates between 8 AM and 2 PM (part of the light period) calculated based on the weight changes shown in **C**; Day 1 corresponds to 2023-11-23 and Day 10 to 2023-12-02. Two scales were connected to each Raspberry Pi Pico W, and the scales were calibrated before each experiment. With this setup, the price of the parts was about US$25 per scale.

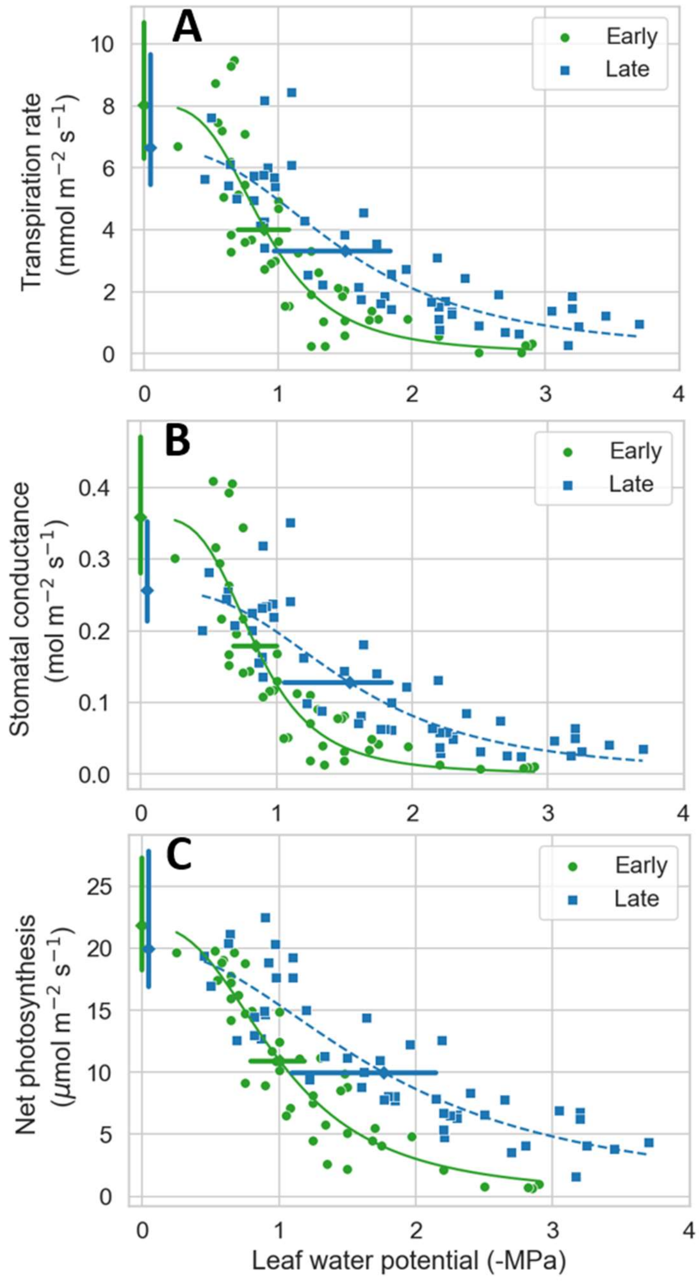

Figure S2. Leaf transpiration rate (**A**), stomatal conductance (**B**), and net photosynthesis (**C**) in response to decreases in leaf water potential (Ψ_l) for early and late leaves of *Artemisia tridentata* seedlings. Data fitted to a sigmoidal function (Equation 2), vertical lines indicate 95% CI for maximal values for Tr, g_s , or NP, and horizontal lines 95% CI for the water potentials at half of these values (Ψ50).

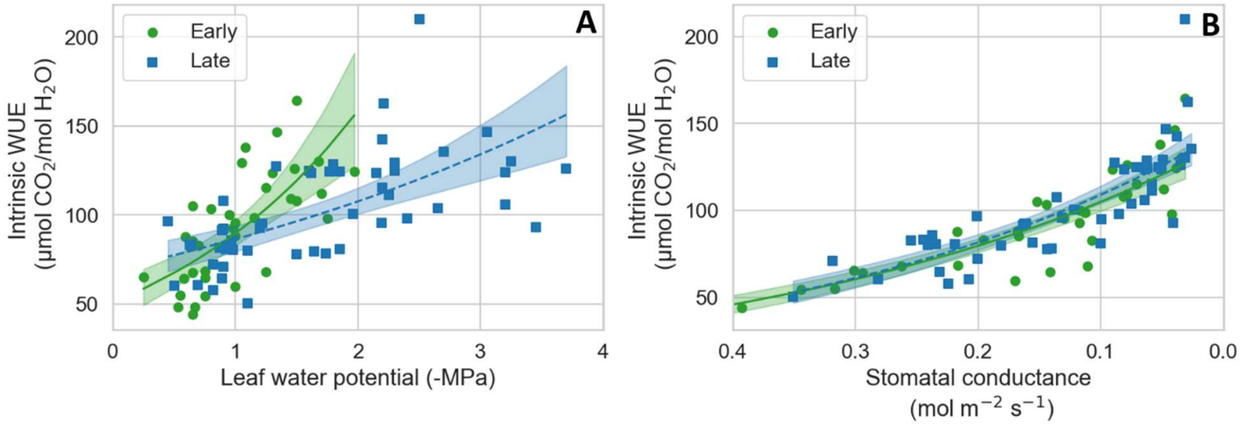

Figure S3. Intrinsic water use efficiency in response to decreasing leaf water potentials (**A**) and stomatal conductance (**B**) for early leaves in three-month-old, and late leaves in twelve-month-old *Artemisia tridentata* seedlings. Data fitted to a generalized linear model with a gamma distribution and a log-link function; each line indicates the best fit and its 95% CI.

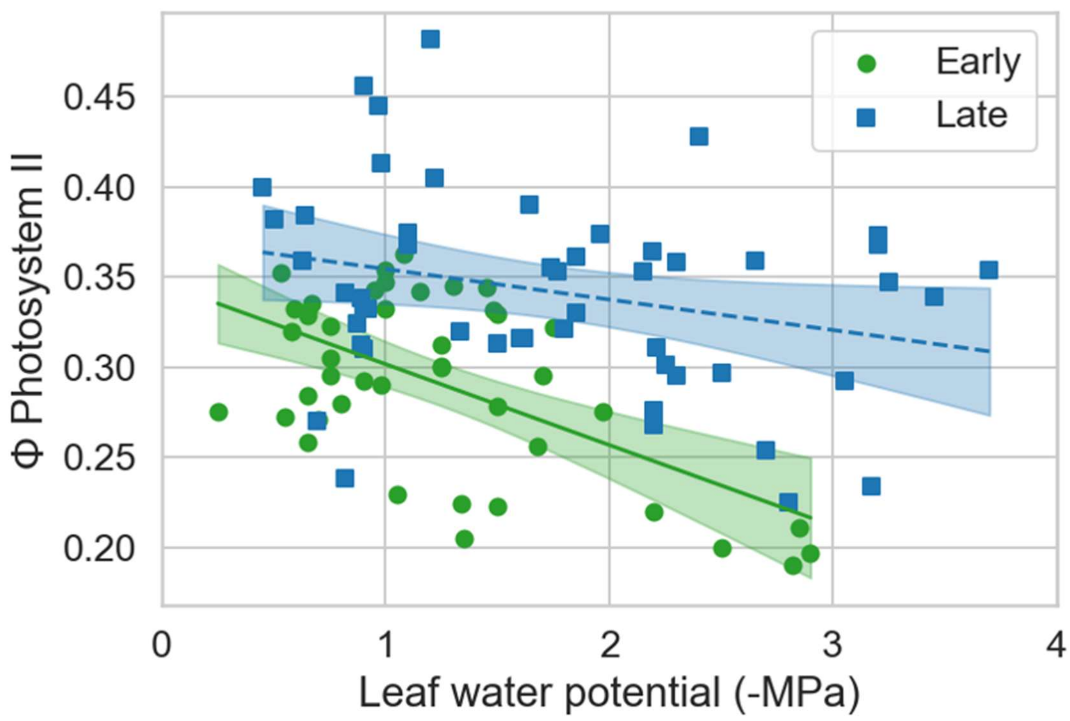

Figure S4. Change in photosystem II operating efficiency (ΦPSII) as a function of leaf water potential (Ψ_l) for early and late leaves of *Artemisia tridentata* seedlings. Data fitted to a generalized linear model with a gamma distribution and a log-link function; each line indicates the best fit and its 95% CI.

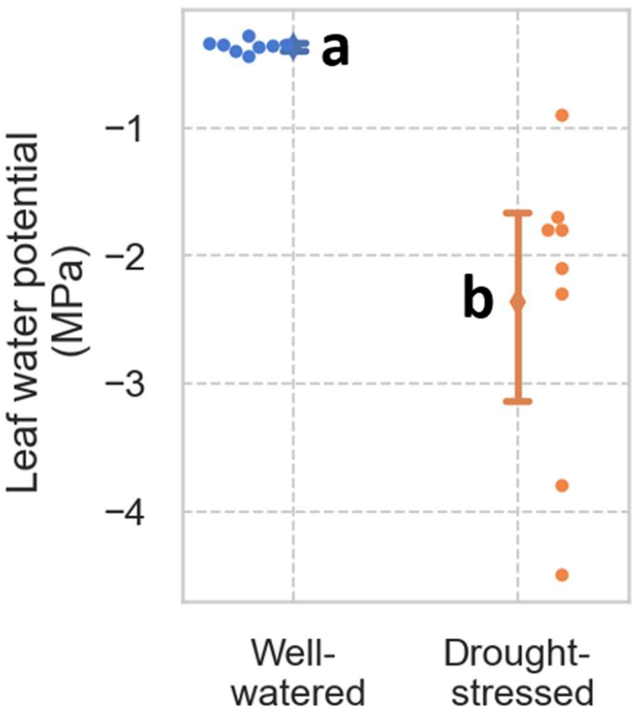

Figure S5. Water potential of three-month-old *Artemisia tridentata* seedlings that were kept well-watered or drought-stressed until whole plant transpiration declined to an average of about 10% of that initially observed. For the drought-stressed seedlings, withholding watering was initiated when the seedlings were two months old. The stress consisted of three cycles of withholding watering for approximately 10 days, followed by rewatering. The Ψ_l of the drought-stressed seedlings was measured at the end of the third drying period.
